# Structure-guided engineering of a flavin-containing monooxygenase for the efficient production of indirubin

**DOI:** 10.1186/s40643-022-00559-7

**Published:** 2022-06-27

**Authors:** Bing-Yao Sun, Hua-Lu Sui, Zi-Wei Liu, Xin-Yi Tao, Bei Gao, Ming Zhao, Yu-Shu Ma, Jian Zhao, Min Liu, Feng-Qing Wang, Dong-Zhi Wei

**Affiliations:** State Key Lab of Bioreactor Engineering, Newworld Institute of Biotechnology, East China University of Science and Technology, Shanghai, 200237 China

**Keywords:** Flavin-containing monooxygenase, Indirubin, Loop region, Substrate tunnel, Structure-guided enzyme engineering, Microbial synthesis

## Abstract

Indirubin is a bisindole compound for the treatment of chronic myelocytic leukemia. Here, we presented a structure-guided method to improve the activity of a flavin-containing monooxygenase (bFMO) for the efficient production of indirubin in *Escherichia coli*. A flexible loop interlocked with the active pocket through a helix and the substrate tunnel rather than the active pocket in bFMO were identified to be two reconfigurable structures to improve its activity, resulting in K223R and N291T mutants with enhanced catalytic activity by 2.5- and 2.0-fold, respectively. A combined modification at the two regions (K223R/D317S) achieved a 6.6-fold improvement in catalytic efficiency (*k*_cat_/*K*_m_) due to enhancing *π*–*π* stacking interactions stabilization. Finally, an engineered *E. coli* strain was constructed by metabolic engineering, which could produce 860.7 mg/L (18 mg/L/h) indirubin, the highest yield ever reported. This work provides new insight into the redesign of FMOs to boost their activities and an efficient approach to produce indirubin.

**Graphical Abstract:**

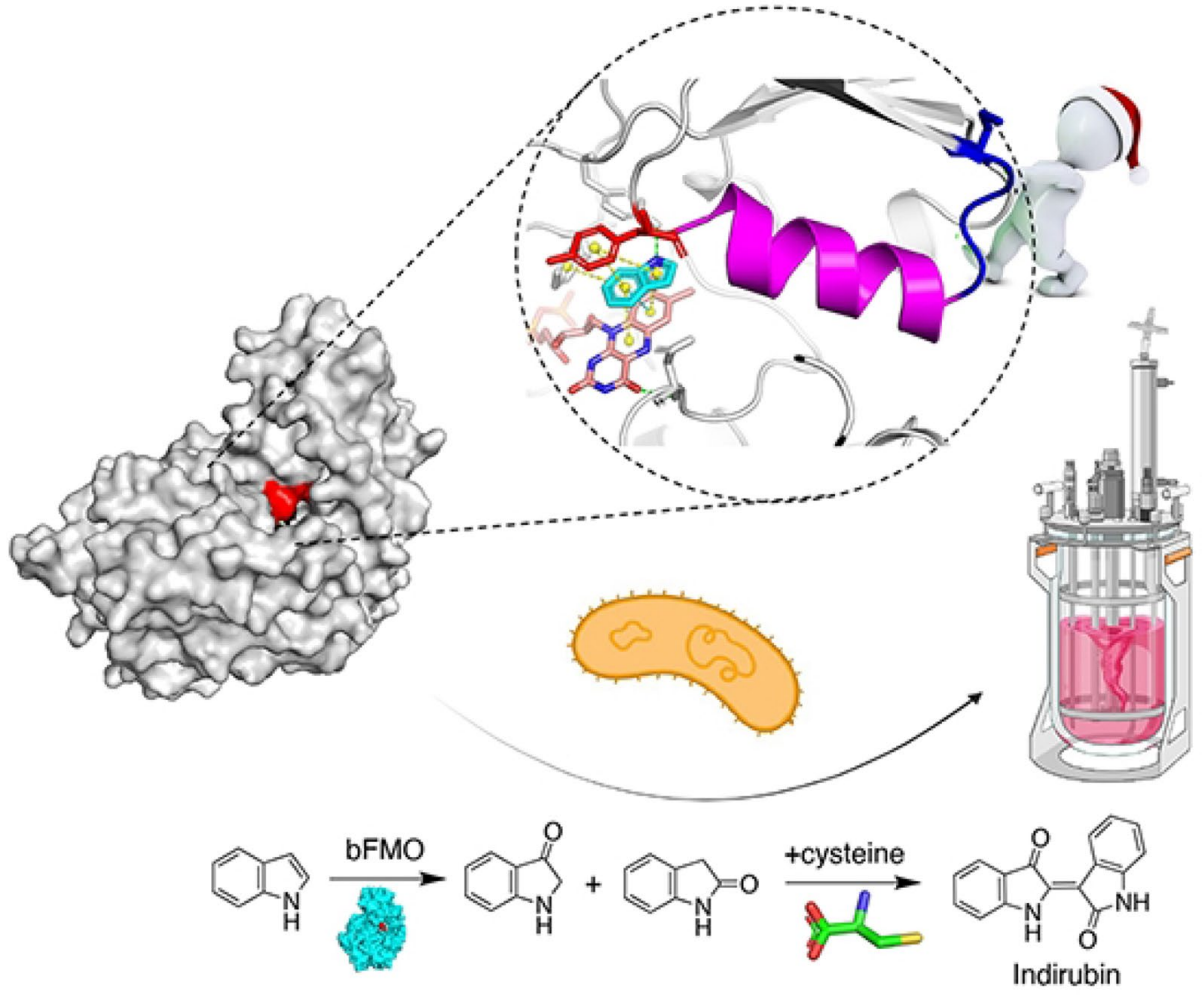

**Supplementary Information:**

The online version contains supplementary material available at 10.1186/s40643-022-00559-7.

## Introduction

Traditional Chinese medicine (TCM) is used worldwide for the treatment of diverse diseases (Efferth et al. 2007). However, to recover TCM from plants usually require complex extraction and purification processes, which often involves the use of large amounts of cultivated plants and hazardous organic solvent due to the low active ingredients with complex compositions (Anastas and Eghbali 2010). Thus, the demand for renewable and ecologically friendly producing process has become very critical and urgent during the past decades. Indirubin, a natural indole alkaloid, is a major active ingredient of the widely used TCM “Danggui Longhui Wan” in the clinical treatment of chronic myelocytic leukemia (Hoessel et al. 1999). It has also been identified as a highly effective inhibitor of cyclin-dependent kinases (CDKs) (Malumbres and Barbacid 2009) and glycogen synthase kinase-3*β* (GSK-3*β*) (Leclerc et al. 2001) and exhibits promise in treating some diseases, including cancer and Alzheimer’s disease (Hoessel et al. 1999; Nam et al. 2005; Seyed et al. 2016). Therefore, indirubin with medicinal grade purity is a popular agent. Direct extraction from herbs, such as the roots or leaves of *Isatis indigotica*, is currently a major way to produce indirubin in the industry (Du et al. 2018). It is not a sustainable process because of consuming a great amount of organic solvent and the recovery of which is only 0.026% from dry *Isatis indigotica* leaves (Lü et al. 2012). Nowadays, with the rapid advances of synthetic biology, harnessing microorganisms to synthesize indirubin provides a promising solution for the sustainable production of indirubin (Kim et al. 2019).

For the biosynthesis of indirubin, a key hydroxylation step is responsible for the conversion of indole to indirubin. Several biocatalysts, such as human cytochrome P450 mutants (Gillam et al. 2000), cytochrome P450 BM-3 mutants (Li et al. 2000), naphthalene dioxygenase (NDO) (Berry et al. 2002), toluene ortho-monooxygenase (TOM) variants (A113V, V106P, V106Q/A113G) (Rui et al. 2005), and toluene-4-monooxygenase (T4MO) (McClay et al. 2005) have been reported to be effective in the conversion of indole to indirubin. Unfortunately, indirubin is only a minor byproduct of the reaction, and indigo, an isomer of indirubin, is usually the major product. Most of the previous studies mainly focused on indigo production because of the low yield and inefficiency of indirubin production (Latimer et al. 2020). Attempts have been carried out to improve the production of indirubin by selecting and engineering new enzymes (Choi et al. 2003) and metabolic pathways (Du et al. 2018; Hu et al. 2010). A flavin-containing monooxygenase from *Methylophaga aminisulfidivorans* (bFMO) has been considered a promising enzyme due to its high activity in converting indole to produce indirubin and indigo (Choi et al. 2003). Using a whole-cell catalyst of bFMO, 5 mg/L indirubin and 920 mg/L indigo were obtained from the conversion of l-tryptophan (Han et al. 2008), and 223.6 mg/L indirubin was produced by supplementing 0.36 g/L cysteine to inhibit the production of indigo (Han et al. 2013). An engineered *E. coli* was constructed using the terpenoid cyclase and the flavin-reducing enzyme to produce 250.7 mg/L indirubin, which is the highest production of indirubin ever reported (Yin et al. 2021).

**Fig. 1:**
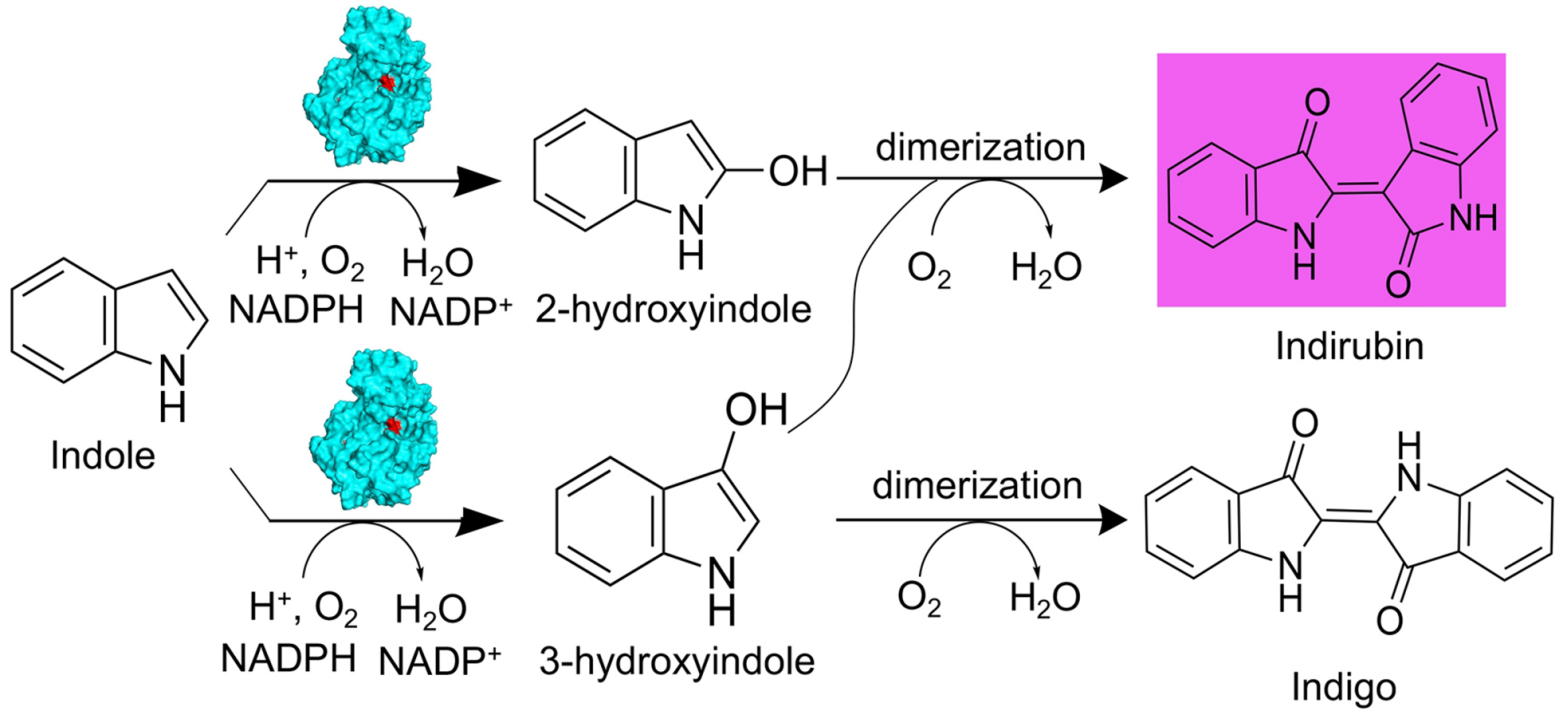
Scheme of the bFMO-mediated catalytic reaction

Flavin-containing monooxygenases (FMOs) are the most important monooxygenase systems involved in xenobiotic metabolism in humans after cytochromes P450, which are also very powerful biotechnological tools and attractive model systems to study the flavin-mediated oxygenation, playing a variety of key physiological roles (Zhang et al. 2020). FMOs belong to the flavoenzyme class of single component flavoprotein monooxygenases (van Berkel et al. 2006); FMOs use equivalent NADPH cofactor to reduce the FAD cofactor, forming FADH_2_, then in turn can react with molecular oxygen and then catalyze the oxidation of a wide range of substrates, such as indole (Alfieri et al. 2008). Bacterial bFMO was found to be able to catalyze the transformation of indole into 2-hydroxyindole and 3-hydroxyindole using the reducing power of NADPH in the presence of oxygen (Fig. 1) (Alfieri et al. 2008). Indigo was produced from the dimerization of two 3-hydroxyindole molecules, whereas indirubin was generated from the dimerization of 2-hydroxyindole and 3-hydroxyindole (Berry et al. 2002). Kim et al*.* elucidated the underlying mechanisms by which cysteine reacted with 3-hydroxyindole to generate 2-cysteinylindoleninone, inhibiting the dimerization of 3-hydroxyindole to generate indigo, thus forming indirubin as the major product (Kim et al. 2019).

Alfieri et al*.* resolved the crystal structure of bFMO (PDB:2VQ7). (Alfieri et al. 2008) Cho et al*.* described the crystal structures of wild-type bFMO (2XVE) and bFMO^Y207S^ with the substrate indole (2XVJ) and revealed that indole and NADPH/NADP^+^ can compete the space of the key amino acid Y207 above FAD in the active site of the enzyme (Cho et al. 2011). The Y207S mutant fails to catalyze the production of indigo in *E. coli* under various concentrations of indole, confirming that Y207 is a key residue in indole oxygenation by bFMO (Cho et al. 2011). Although bFMO is the best enzyme for the oxidation of indole to produce indirubin reported thus far, the low catalytic activity still limits its production efficiency. Loncar et al*.* used the FRESCO protocol to achieve higher thermostability and showed that the *k*_cat_ for indole to produce indigo could be improved 1.28-fold by screening a C78I mutant (Loncar et al. 2019). Nevertheless, the underlying molecular mechanism of FMO for catalyzing indole to generate indirubin remains elusive, and it is challenging to improve the enzyme activity and further enhance indirubin production. To our limited knowledge, restricted work on enzyme engineering of bFMO has been conducted to achieve the green and efficient production of indirubin. Moreover, for the highly efficient approach of directed evolution, a suitable high-throughput screening system to select positive mutants for indirubin production is still a bottleneck.

**Fig. 2:**
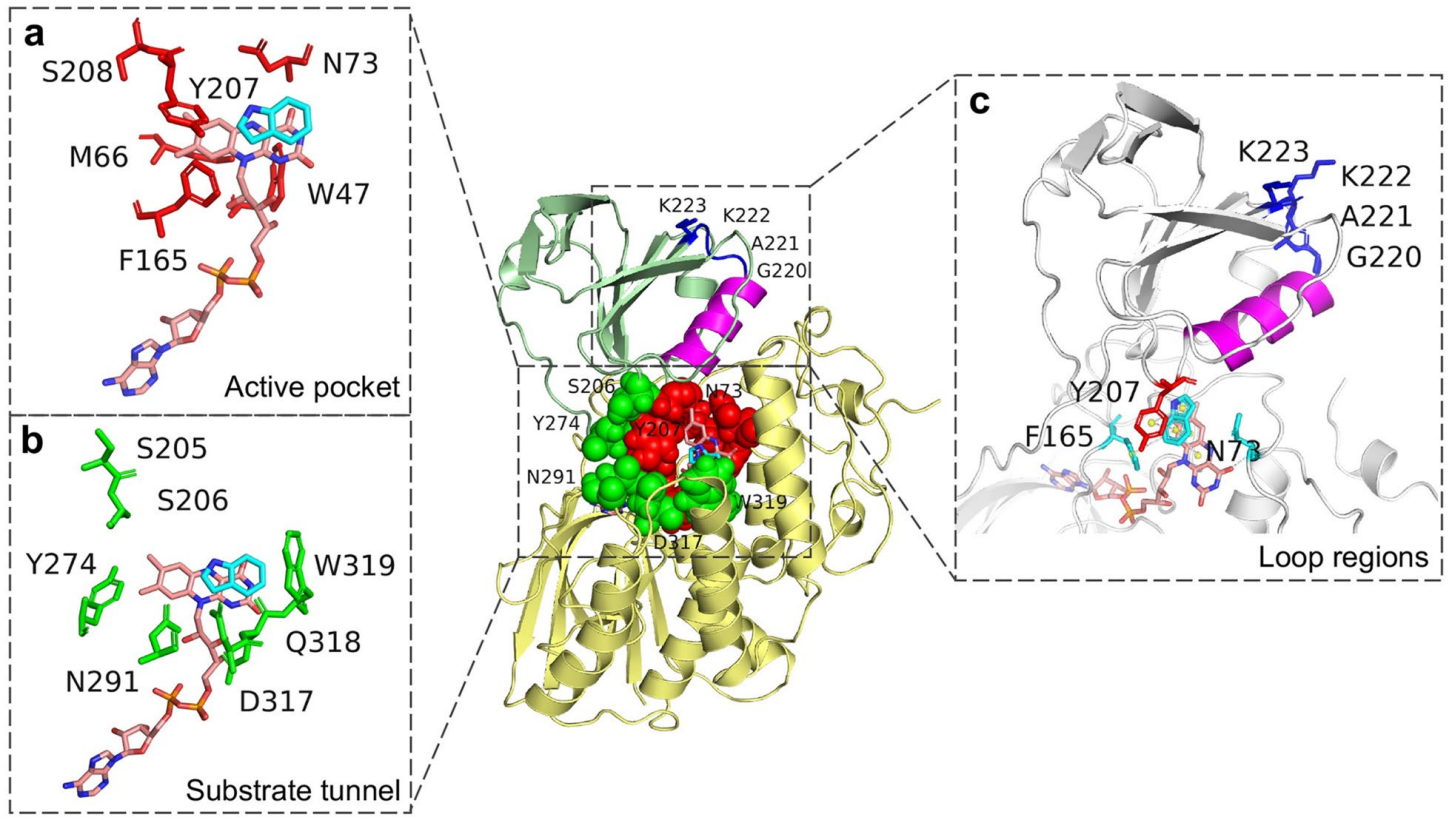
Docking analysis of bFMO–indole complex, showing the location of active pocket residues (red), substrate tunnel residues (green) and the loop (blue) connected the α4 helix (purple), which have been targeted for mutagenesis. The FAD-binding domain (residues 1–164 and residues 276–445) is shown in yellow; the NADPH-binding domain (residues 165–275) is shown in green. The residues of the substrate tunnel (green) and the active pocket (red) are represented as spheres. The cofactors FAD (orange) and substrate indole (cyan) are represented as sticks. **a** Six residues located at active pocket within 8 Å with the indole, shown in red sticks. **b** Seven residues located at substrate tunnel, shown in green sticks. **c** Loop (residues 220–223, blue) connected with the α4 helix (purple). The π–π stacking interaction in the active pocket of bFMO, as shown in yellow lines

In the present work, computational simulations and experimental approaches were combined to improve the in vivo and in vitro catalytic activity of bFMO for enhanced production of indirubin. The structure-guided directed evolution of bFMO was conducted to maximize the likelihood of identifying useful mutations based on computational analysis of enzyme–substrate docking and molecular dynamics (MD) simulations. First, the active pocket of bFMO was redesigned by site saturated mutation. Unfortunately, no positive mutant was obtained. Targeting flexible structural elements, such as loop regions or substrate tunnels, has been shown to influence the dominant conformations of enzymes, which can be advantageous to efficient catalysis (Henzler-Wildman et al. 2007). The structural regions of different functions for stabilizing the interaction of indole and FAD and the roles of the flexible loop regions and substrate tunnel modification in bFMO are not fully understood. Based on the structural analysis, we found that the *α*4 helix was closely associated with the key amino acid of Y207. It was assumed that conformational changes could be generated in space to shift the tyrosine side chain of Y207 in the active site when substrate indole docked into the active pocket. A hypothesis facilitated by a structure-guided engineering strategy was proposed: modifying the flexible residues of a small loop beside the *α*4 helix (Fig. 2) to precisely adjust the position of Y207, which might affect the stability of indole and FAD in the hydroxylation reaction of bFMO. Moreover, a high-throughput screening method for selecting bFMO mutants with higher catalytic activity for indirubin yield was developed by adding cysteine. Finally, an efficient indirubin-producing strain was obtained with the highest yield ever reported and the output of indirubin in 200 L fermentation broth was equivalent to the harvest of 1-acre *Isatis indigotica*, showing an economical way for the sustainable production of indirubin.

## Materials and methods

### Strains and materials

The recombinant strains harboring *bFMO* from *Methylophaga aminosulfidovorans* were constructed using plasmid pET28a stocked in our lab. *Escherichia coli* BL21(DE3) was purchased from TransGen Biotech Co. (Beijing, China). (Choi et al. 2003) Indigo, indirubin, and indole were purchased from Aladdin Co., China. *Dpn* I was purchased from Thermo Fisher (Thermo Fisher Scientific, USA). KOD One™ PCR Master Mix was purchased from TOYOBO (TOYOBO Life Science, Japan). Hieff clone Plus One Step Cloning kit was purchased from YEASEN (YEASEN Biotechnology Co., Shanghai, China). All the other chemicals were commercially available. The tryptophan medium contained 5 g/L yeast extract, 10 g/L NaCl, 2 g/L tryptophan. The cysteine medium contained 5 g/L yeast extract, 10 g/L NaCl, 2 g/L tryptophan, 0.36 g/L cysteine.

### Molecular docking

The crystal structure of bFMO from *Methylophaga aminosulfidovorans* was downloaded from the Protein Data Bank (PDB: 2XVE) (Cho et al. 2011) and structures of the mutants obtained by single point mutations were used as receptors. Molecular docking was performed by Autodock 4.2, and the ligand indole was obtained from the ZINC database (Morris et al. 2009). While the ligand was completely flexible, protein residues were kept rigid except from setting N73, F165 and Y207 as flexible residues. For indole, the search space for modelling was set as follows: Grid box center (*x* = 44, *y* = 42, *z* = 43); Gride box size (*x* = 40, *y* = 40, *z* = 40). The docking performance was conducted with genetic algorithm parameters and the number of runs 100. Best binding modes were visualized by PyMOL.

### Site-directed saturation mutagenesis

The bFMO variants were created by PCR using the *bFMO* gene as the template. Oligonucleotide primers used for the generation of mutants were listed in Additional file 1: Table S2. The PCR mixture (total volume 50 μL) contained: template plasmid (1 μL, 20 ng/μL), 2*KOD One™ PCR Master Mix (25 μL), forward and reverse primers (1 μL, 10 μM each), and ddH_2_O (22 μL). The PCR mixture was first subjected to 94 °C for 4 min, followed by 30 cycles of denaturing at 98 °C for 10 s, annealing at 55 °C for 30 s, and elongation at 68 °C for 2 min. A final extension step at 68 °C for 10 min was conducted at the end of the PCR reaction. 2 μL *Dpn*I enzyme was then added to the PCR reaction mixture to digest the template plasmid pET28a–bFMO at 37 °C for 1 h; *Dpn*I-digested PCR products were finally self-ligated by Hieff clone Enzyme Premix. The resulting reaction mixtures were transformed into *E. coli* BL21(DE3) competent cell according to the Laboratory Molecular Cloning Manual.

### High-throughput screening methods

To achieve rapid and efficient screening, it was necessary to establish a high-throughput screening (HTS) method for bFMO. The HTS method was constructed based on different fermentation media (tryptophan medium or cysteine medium) (Kim et al. 2019). Clones were picked based on the dark-colored and introduced into 96-deep-well plates that containing 300 μL LB culture medium with 100 ng/μL kanamycin. After being cultured overnight, 10% of the sub-cultures were transferred to 96-deep-well plates with additional tryptophan or cysteine as the secondary plate with the same inoculum amount. For protein expression, IPTG was added to a final concentration of 0.2 mM after cultivation at 37 °C for 2 h. After incubating at 30 °C for another 24 h with shaking at 200 rpm, cells were harvested by centrifugation at 2000×*g* and 4 °C for 10 min and the pigment can be extracted by DMSO and detected at 540 nm (indirubin) and 620 nm (indigo) with the imaging reader, respectively. In this way, we could detect both products simultaneously and avoid the presence of indigo affecting the detection of indirubin. Mutants with higher production of indirubin than the control were selected for further shaking flask fermentation followed by HPLC analysis.

### Analytic methods

The production of indigo and indirubin was determined by HPLC analysis using (C18, 250 mm × 4.6 mm) (Agilent Technologies, Ltd, USA) with an eluent flow rate of 0.8 mL min^−1^, and monitored at 289 nm with the mobile phase of methanol and water (80:20, v/v).

### Expression and purification of bFMO

Total 10 μL fermentation solution was used to inoculate 5 mL LB medium (containing 50 μg/mL kanamycin) and incubated at 37 °C and 220 rpm overnight. The overnight sub-cultures were transferred to 200 mL LB (1%, v/v) containing 50 μg/mL kanamycin and incubated at 37 °C and 200 rpm until OD_600_ reached 0.6–0.8. Then IPTG (final concentration of 0.2 mM) was added to induce bFMO expression and the culture was then incubated at 18 °C for another 24 h with shaking at 200 rpm. Cells were harvested by centrifugation at 8000×*g* and 4 °C for 10 min and the supernatant was discarded. The cells were resuspended in the buffer (20 mM phosphate buffer; pH 7.4) and the target proteins inside the cells were released by ultrasonication. The cell debris was removed by centrifugation at 8000×*g* for 30 min at 4 °C.

### Determination of enzyme activity and kinetic constants

FMO activity was determined spectrophotometrically using indole as the substrate. The assay mixture (1 mL) contained 5 mM indole and 0.2 mM NADPH dissolved in 20 mM PBS buffer (pH 7.4), and purified enzyme with a final concentration of 0.1 mg/mL was added to the mixture. The reaction rates of the enzyme were monitored by following NADPH oxidation at 340 nm. Protein concentrations were determined at 595 nm by the Bradford method (Bradford 1976). The kinetic constants of wide-type bFMO and mutants were measured under standard assay condition with various substrate indole concentrations (0.1, 0.3, 0.5, 0.7, 1, 2, 3, 4, 5, 6, 8 and 10 mM) and various NADPH concentrations (0.01, 0.02, 0.03, 0.04, 0.05, 0.06, 0.08, 0.1, 0.15, 0.2, 0.25 and 0.3 mM). The data were fitted to the Michaelis–Menten equation using nonlinear regression analysis with the Origin 2018 software. One unit of the enzyme activity was defined as the enzyme amount that consuming 1 μmol NADPH per minute under standard assay conditions. All measurements were conducted in triplicate in the present study and data were means ± standard deviations from three replications.

### Molecular dynamics simulation

Molecular dynamics simulation was performed using GROMACS 2019 (http://www.gromacs.org/) with the amber99sb-ildn force field (http://ambermd.org/) (Apol et al. 2013) and the TIP3P model was used for water molecules. The force field parameters of small molecules were generated by the acpype.py script in AmberTools2018 (Batista et al. 2006), which was introduced in the GROMACS tutorial for protein–ligand system (http://www.mdtutorials.com/gmx/complex/index.html). First, energy minimization was conducted with the steepest descent method by minimizing the energy of the systems, aiming for the close contact between atoms. After the optimization, each system was heated from 0 to 300 K gradually under the NVT ensemble for 100 ps, followed by 100 ps NPT ensemble. The V-rescale temperature coupling method was used to control the simulated temperature at 300 K and the Berendsen method was used to control the pressure at 1.0 atm. During the MD simulation, the LINCS algorithm was applied to constrain all hydrogen bonds and the step size was 2 fs. The electrostatic interaction was calculated with the PME (Particle-Mesh Ewald) method with a cut-off value of 1.2 nm. The cutoff of non-bonded interactions was 10 Å.

### Fed-batch fermentation

For fed-batch fermentation, the single clone of W3 strain was transferred to 5 mL LB medium and was cultivated at 37 °C for approximately 18 h. The second seed cultures were transferred to 500 mL baffled flask carrying 100 mL LB medium and were incubated at 37 °C for 18 h with 200 rpm agitation. The seed cultures were inoculated into a 5-L jar fermenter (Bailun bio, China Shanghai) with 2.4 L of initial batch fermentation medium. The initial batch fermentation was started using modified fed-batch fermentation medium as follows: 5 g/L glucose, 20 g/L yeast extract, 8 g/L tryptone, 1 g/L MgSO_4_, 2 g/L KH_2_PO_4_, 4 g/L K_2_HPO_4_, 1 g/L (NH4)_2_SO_4_, 10 g/L L-tryphan, 5 g/L l-cysteine. The glucose concentration was maintained below 2 g/L by supplementing 300 g/L of glucose. The temperature was controlled at 30℃, and the initial agitation was set to be 200 rpm and increased to maximally 800 rpm depending on the dissolved oxygen level, which was maintained above 30% saturation by controlling the aeration (the initial airflow rate of 2 vvm) and agitation speed. The pH of the fermentation medium was maintained at 6.9–7.1 by automatic feeding of NH_4_OH (25%, v/v).

## Results and discussion

### Structure-guided analysis of bFMO for indirubin production

An in-depth understanding of the enzyme structure–function relationship and enzyme–substrate interaction has important practical and fundamental significance for the rational design of enzymes (Kreß et al. 2018; Liu et al. 2020). For mutants generated by protein engineering, molecular docking and dynamics simulations have been broadly employed in the structure-guided design to improve the catalytic efficiency of enzymes (Dong et al. 2020; Loncar et al. 2019).

As shown in Fig. 2, the crystal structure of bFMO is made up of two distinct domains: a large FAD-binding domain (residues 1–164 and 276–447) and a smaller NADPH-binding domain (residues 165–275) (Alfieri et al. 2008). Cho et al. determined the crystal structure of wild-type bFMO (PDB ID: 2XVE) and a Y207S substitution mutant of bFMO (bFMO^Y207S^) with indole (PDB ID: 2XVJ), which lacks indole oxygenation activity (Cho et al. 2011). To analyze the interaction between indole and wild-type bFMO and find the key residues participating in the oxygenation process of indole, semiflexible molecule docking was conducted based on the crystal structures of wild-type bFMO (PDB ID: 2XVE) (Cho et al. 2011) by Autodock 4.2. Considering that the residues Y207, N73, and F165 could contribute to the interaction with the isoalloxazine ring of FAD (Additional file 1: Fig. S1) (Cho et al. 2011), they were set to be flexible. Six key residues (i.e.*,* W47, M66, N73, F165, Y207 and S208) in the active pocket were selected for site saturation mutagenesis (Fig. 2a). However, most mutations showed decreased catalytic efficiency for indirubin production (data not shown), especially almost all of the mutants of the Y207 and N73 sites were dramatically colorless. By comparing the crystal structures of wild-type bFMO and bFMO with indole, it was found that the tyrosine ring of Y207 in native bFMO occupies the space of indole binding position above the isoalloxazine ring of FAD, thus might prevent access of indole into the active pocket (Additional file 1: Fig. S1) (Cho et al. 2011).

Enzymes can be modified through protein engineering to improve their activity, which is generally focused on the modification of active sites in the catalytic active pocket (Tan et al. 2021). Nevertheless, the amino acids lining the active pocket are generally conserved, such as the key residue Y207, the mutation of which results in the loss of the indole oxygenation activity of bFMO. Y207 and N73 residues, located at the intersection between the FAD- and NADPH-binding domains, have been revealed to have an allosteric effect, which can trigger a strong domain movement of bFMO (Cho et al. 2011). The tyrosine ring of Y207 is positioned near FAD and participates in hydrogen bonding with the amidogen of N73 directly (Alfieri et al. 2008). Moreover, the highly conserved asparagine residue N73 forms hydrogen bonds with the isoalloxazine of flavin, which could contribute to stabilization through polar interactions (Alfieri et al. 2008). Thus, it was indicated that these two conserved residues played important roles in the reaction, and their mutations could be detrimental to the oxygenation activity of bFMO. Promoted by these initial results, we then sought to enhance the catalytic efficiency of bFMO by targeting the flexible parts of enzymes, such as loop regions or substrate tunnels, and a structure-guided engineering strategy focusing on precisely adjusting the position of Y207 was applied for this purpose.

### High-throughput screening of bFMO variants for indirubin production

**Fig. 3:**
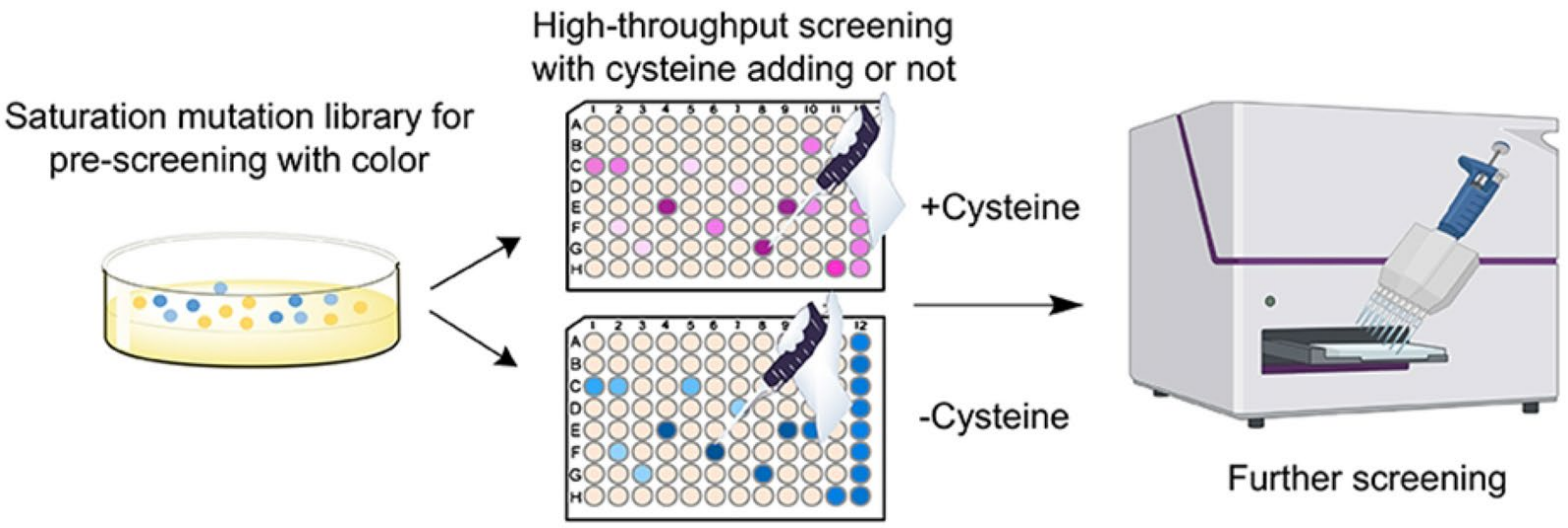
Schematic representation of the high-throughput screening process for the activity assay of indirubin. After pre-screening with color, cysteine was added to the secondary culture to obtain the pure indirubin, and then absorbance was measured by UV spectrophotometry microplate reader

To facilitate engineering of bFMO with improved catalytic activity, establishment of a high-throughput screening method for positive variants from large mutagenesis libraries is crucial. However, for bFMO, indirubin is synthesized as a byproduct of indigo, which is a major obstacle for the selection of potential positive mutants for indirubin production (Choi et al. 2003). Kim et al. elucidated the underlying mechanisms involved in shifting the product selectivity from indigo to indirubin by supplementation with cysteine for bFMO (Kim et al. 2019). Thus, we developed a novel high-throughput screening method for the mutagenesis and selection of positive bFMO mutants with improved catalytic efficiency for indirubin production, as shown in Fig. 3. A primary library was constructed by saturated mutation of the hotspot residues, screening the deep-color clones, and then transferring them to 96-well plates. Through color screening, the pure indirubin product was obtained by adding cysteine into the medium when the 96-well plate was transferred, which could be easily detected and avoid interference with the presence of indigo (Additional file 1: Fig. S2). This approach provided an efficient high-throughput screening method for FMOs via the selection of positive mutants for indirubin production.

### Modification of a flexible loop region (residues 220–223)

The functional hotspots of flexible structural elements, such as loop regions and substrate tunnels, beyond the active pocket, has gained the increasing interest in unlocking the potential of enzyme (Kreß et al. 2018; Meng et al. 2021; Rapp et al. 2021). Flexible loops connecting the main secondary structures (*α*-helices and *β*-strands) primarily have an effect on conformational changes in the interaction with catalytic residues and the binding active pocket (Liu et al. 2020; Ni et al. 2021). Thus, we aimed to further identify functional hotspots beyond the active sites in the substrate pocket for the creation of smart mutant libraries.

**Fig. 4:**
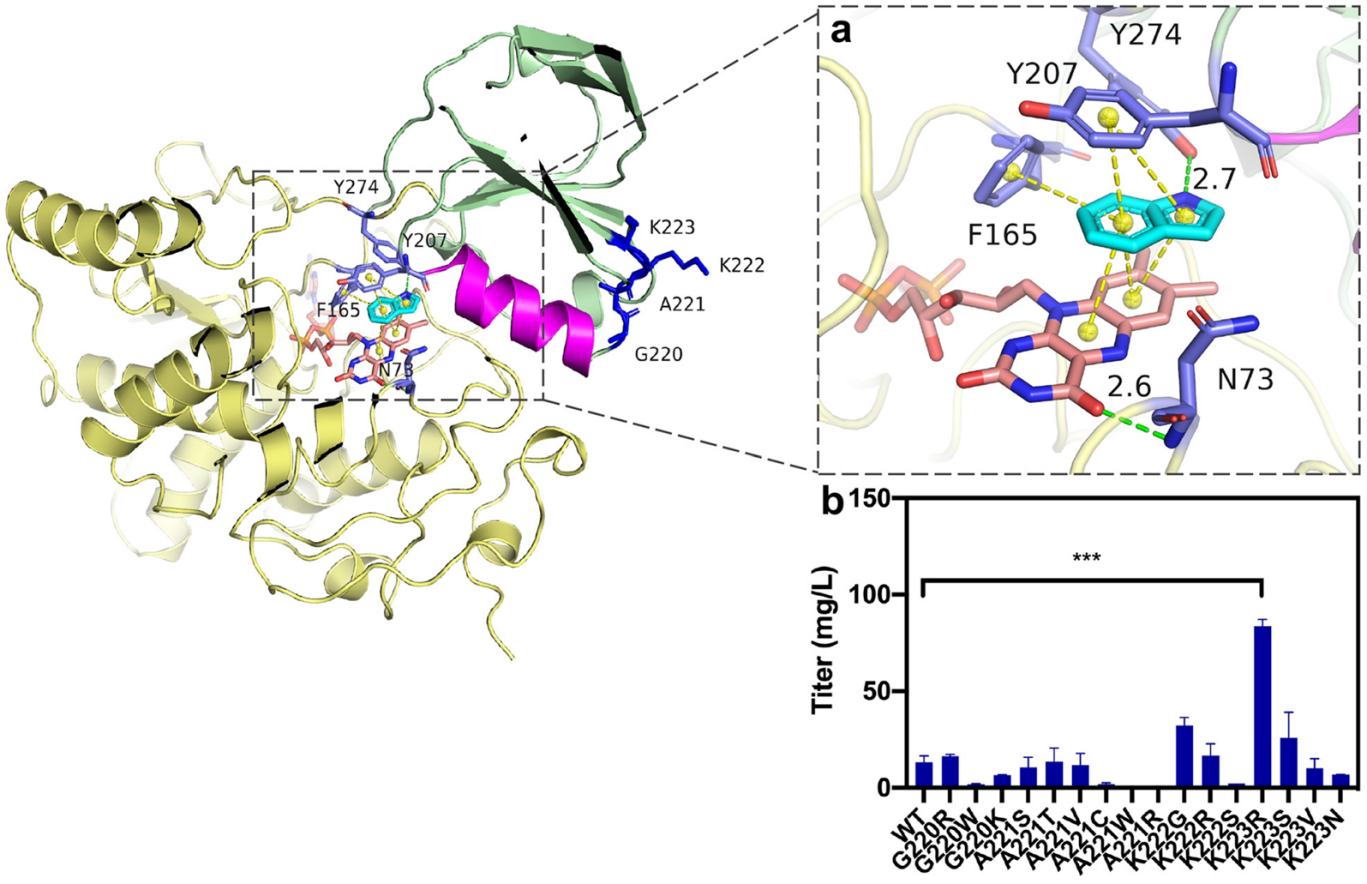
Docking analysis of the bFMO–indole complex, which shows the location of the*α*4 helix (purple) and the loop 220–223 (blue). **a** π–π stacking interactions of Y207–indole–FAD are indicated by yellow dashed lines, and residues in blue sticks. The F165 is also involved in π–π stacking interaction with indole. Green dotted line indicates hydrogen bond between N73 and FAD. **b** Indirubin titer of the mutants of bFMO in vivo. Statistical analysis was performed using the Student’s *t* test (one-tailed; two-sample unequal variance; *p < 0.05, **p < 0.01, ***p < 0.001). All data represent the mean of *n* = 3 biologically independent samples and error bars show the standard deviation

As shown in Fig. 4, the *α*4 helix (residues 208–218), in close proximity to the Y207 residue and the active pocket, is connected with strand *β*11 (residues 241–245) via a small loop (residues 220–223). Considering the potential interaction between Y207 and the *α*4 helix, it was assumed that the reorganization of the *α*4 helix might result in conformational changes of bFMO and affect the catalytic efficacy indirectly via the Y207 residue. Thus, we attempted to modify the flexible residues of G220, A221, K222, and K223 on a small loop (residues 220–223) beside *α*4 helix to precisely adjust the position of Y207, which might change the orientation and proximity of the indole substrate to the FAD group in the *π*–*π* stacking interaction and thus affect the stability of indole and FAD in the hydroxylation reaction of bFMO (Fig. 4a). Thus, site saturation mutagenesis was conducted to identify these four hot-spot residues beside the *α*4 helix to improve the catalytic activity of bFMO. The degenerate NNK codon was subsequently used to construct saturation mutant libraries covering these 4 sites (G220, A221, K222, and K223), including all 20 canonical amino acids. Based on the structure-guided engineering strategy, a small mutant library containing 300–400 colonies for each site was established for these residues. The catalytic efficiency of wild type bFMO and their mutants was assessed by detecting the fermentation broths of mutants and determining the kinetic constants for indole conversion at diverse substrate concentrations (0.1–10 mM) (Fig. S3).

**Fig. 5:**
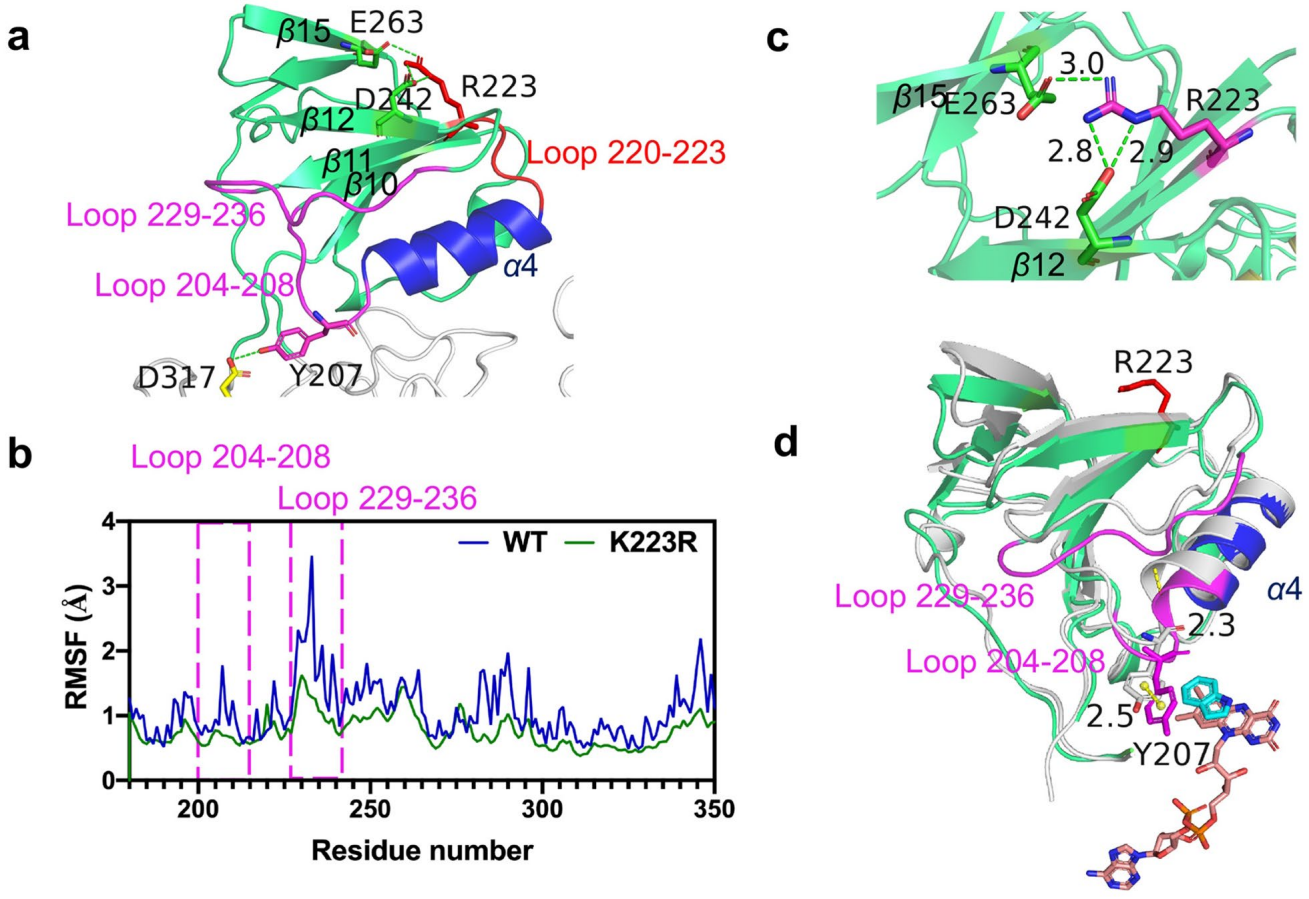
**a** Illusration of the small domain (green) of K223R mutant, which shows the locations of the α4 helix (blue), the loop 229–236 (purple), the loop 204–208 (purple), and the loop 220–223 (red), were displayed in cartoon by PyMOL. **b** RMSF values of wild-type bFMO (blue) and the K223R mutant (green). The RMSF of atomic positions was calculated according to a reference frame in the trajectory, and the RMSF of residues was calculated by averaging the RMSF of atoms of each residue. **c** New hydrogen bond (green dashed lines) generated between R223 with E263 and V200, respectively. R223 (purple), E263 (green) and V200 (green) were displayed as sticks. **d** Movement of α4 helix (blue) in the K223R mutant compared with that of the wild-type bFMO (grey). The distance of the movement was indicated by yellow dashed lines

As a result, the indirubin production of mutant K223R was 5.8-fold higher than that of wild-type bFMO (from 13.3 ± 0.7 mg/L to 76.8 ± 2.9 mg/L), and the mutants K222G (32.3 ± 3.4 mg/L), K222R (16.7 ± 3.0 mg/L), and K223S (26.0 ± 1.7 mg/L) were 2.4-, 1.3- and 2.0-fold higher than that of the control strain containing wild-type bFMO in vivo (Fig. 4b) and the same trend in vitro (Fig. S4), respectively. Molecular dynamics simulations were further conducted with the bFMO–FAD–indole complexes to better reflect the dynamics of the reaction between bFMO and indole. bFMO consists of a large domain and a small domain (*α*4 and *β*9–*β*15) (Cho et al. 2011). The main feature of the small domain is two large *β* sheets comprised of several parallel *β* strands or antiparallel *β* strands and only one helix (*α*4), as shown in Fig. 5a. The structural refinement of the models was performed by energy minimization, and molecular dynamics simulations were conducted for further structure-based analysis. The root-mean-square fluctuation (RMSF) values of *β*10–*α*4 (loop 204–208) and *β*11–*β*12 (loop 229–236) in the K223R mutant were obviously lower than those of the wild-type bFMO (Fig. 5b), indicating the reduced flexibility of these two loops in the K223R variant. When K223 was mutated to arginine, new hydrogen bonding with residue E263 on the *β*15 strand (residues 260–264) and D242 on the *β*12 strand (residues 241–245) was generated with the new R223 residue (Fig. 5c), which led to the *α*4 helix being closer to the active pocket (the *α*4 helix moved 2.3 Å) and increased the stability of the loop regions of 204–208 and 229–236, as shown in Fig. 5d. These two newly generated hydrogen bonds could result in reduced flexibility of the small domain and play roles in maintaining the conformation of the helical structure in the small domain of bFMO, which might be responsible for stabilizing the enzyme–substrate complex and enhancing the catalytic efficiency.

**Fig. 6:**
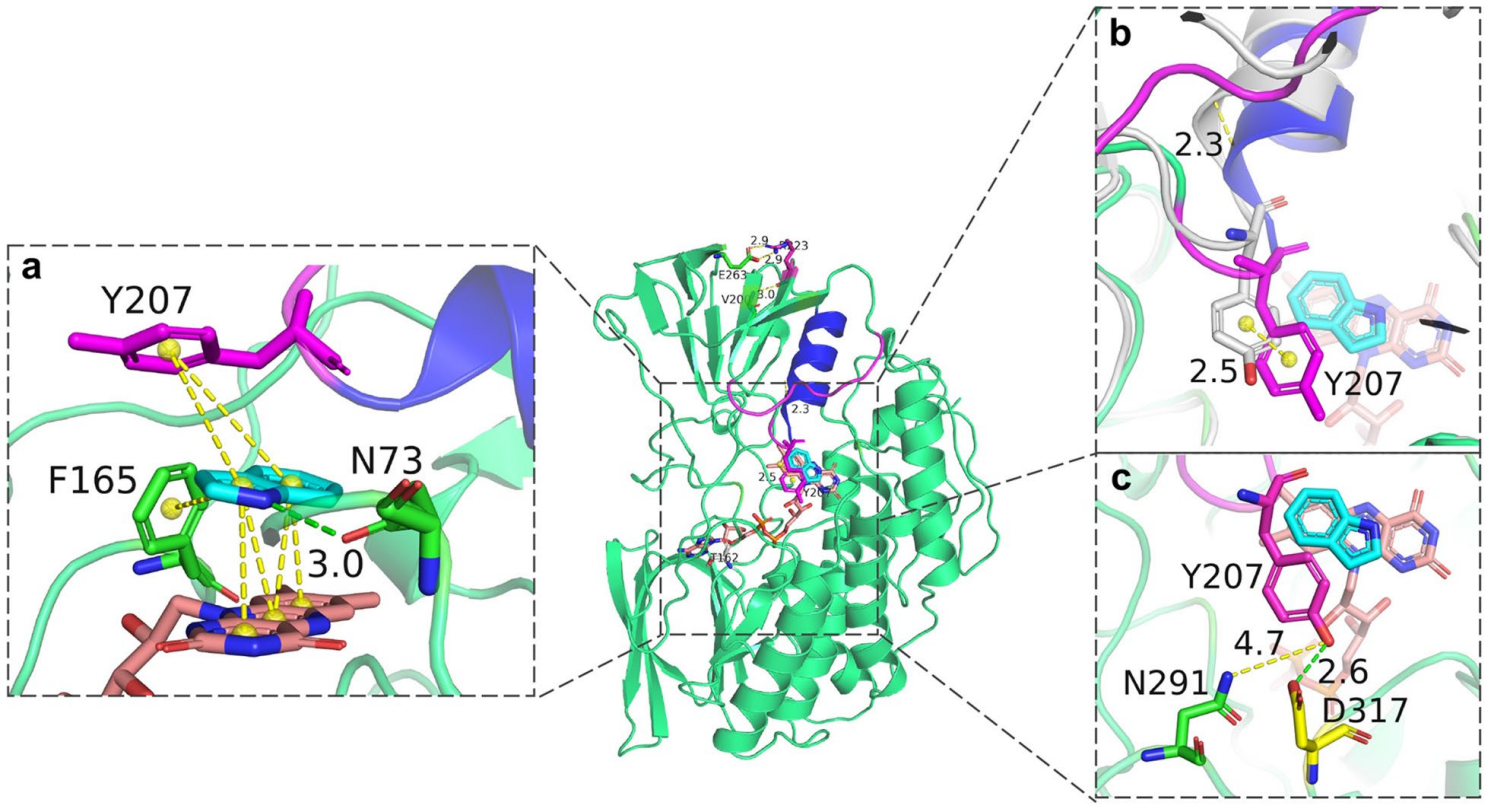
Structure model of the K223R mutant. **a** *π*–*π* stacking interactions between Y207 (or F165) and indole are indicated by yellow dashed lines. The new hydrogen bond was generated between N73 and indole are presented as green dashed line. **b** Comparison of the movement of Y207 (shown in yellow sticks) in the K223R mutant (purple) and in the wild-type bFMO (grey). **c** Distance between residues of Y207 to N291, and Y207 to D317 are represented as yellow lines and green lines, respectively

K223 is distal to the active pocket, which is located on loop 220–223 and connects the *α*4 helix and *β*11 strand. Changes from a distant residue within bFMO could alter the dynamics or conformation of the active pocket via a network of inter-residue interactions (Souffrant et al. 2020). Comparing the two conformations of native bFMO and K223R mutant docked with indole, it was revealed that the movement of *α*4 helix, adjacent to the key residue of Y207, resulted in Y207 shifting around the active sites, i.e., F165 and N73, and affected the *π*–*π* stacking interaction in the active pocket (Fig. 6a). The spatial orientation of the *α*4 helix moved 2.3 Å, which resulted in the tyrosine ring of Y207 moving 2.5 Å toward the indole and the isoalloxazine ring of FAD and contributing to the stability of *π*–*π* stacking interactions between indole and FAD in the K223R mutant model (Fig. 6b). In addition, the multifunctional residue Y207 formed the substrate tunnel with N291, and the distance between them is the bottleneck of the substrate tunnel, which can dramatically affect enzymatic catalysis. The distance between the tyrosine ring of Y207 and the amidogen group of N291 was 4.7 Å, which was longer than that of native bFMO (3.0 Å) (Fig. 6c).

Our data provide insights into the changes in the small domain and point to the loop (residues 220–223) controlling access to the active site, according to which the dynamic conformation changes of the *α*4 helix are essential for catalysis. Thus, besides the residues lining the active pocket of bFMO, regulating residues distant from the active pocket, i.e., the outside loop (residues 220–223) of the *α*4 helix could play important roles in substrate oxygenation. Hu et al*.* conducted similar work in which they mutated the D168 site (located at a loop in close proximity to the key residue V87) far from the active pocket of P450 BM3 and pulled the flexible residue outside the *α* helix to regulate the activity of P450 BM3 (Hu et al. 2010). Since the flexible loop regions could participate in catalytic reactions (Rapp et al. 2021), we hypothesized that the catalytic efficiency of bFMO was enhanced by changing the orientation and proximity of indole to FAD cofactor in the *π*–*π* stacking interaction to increasing the stability of the interaction of indole with FAD, possibly via finely adjusting the position of the conserved residue of Y207. Moreover, it was assumed that the reorganization of the *α*4 helix resulted in conformational changes in the small domain, not only reconstruction of the substrate-binding pocket. This approach provided a model to redesign FMOs for controlling the catalytic activity.

### Modification of the substrate tunnel

**Fig. 7:**
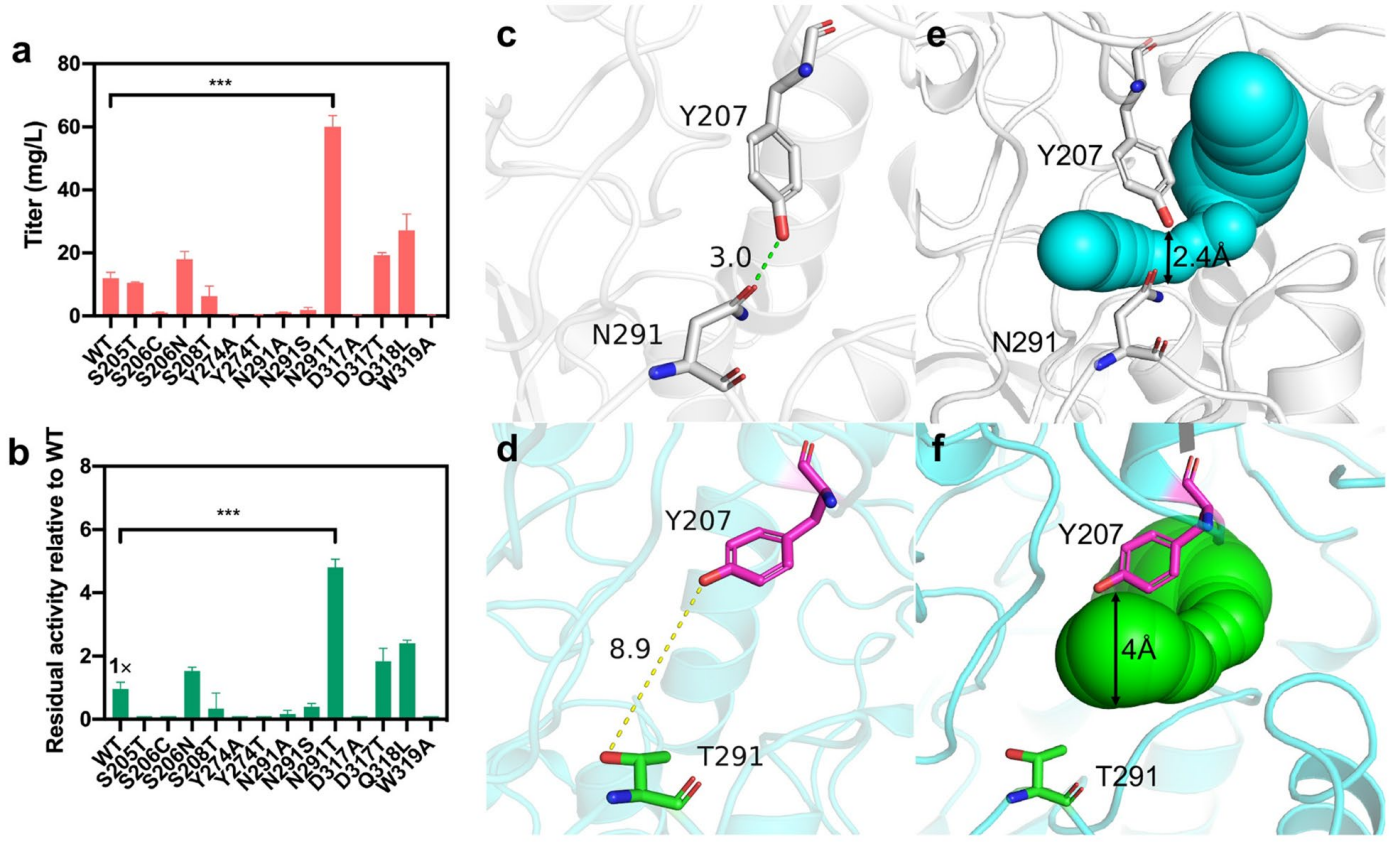
**a** Indirubin titer of various mutants of bFMO in vivo. **b** Enzyme residual activities of various mutants relative to the wild-type (1 ×) toward indole. **c** Hydrogen bond between Y207 and N291 is indicated by green dashed line in wild-type bFMO. **d** Distance between Y207 (purple) and T291 (green) is indicated by yellow dashed lines in N291T mutant. The substrate tunnel shown in balls in wild-type bFMO (**e**) and the N291T mutant (**f**) was identified by CAVER 3.0. Statistical analysis was performed using the Student’s *t* test (one-tailed; two-sample unequal variance; *p < 0.05, **p < 0.01, ***p < 0.001). All data represent the mean of *n* = 3 biologically independent samples and error bars show the standard deviation

From the structure model of bFMO docked with indole (Fig. 2b), seven residues (i.e., S205, S206, Y274, N291, D317, Q318L and W319) in the substrate tunnel surrounding indole within 5–8 Å were considered potentially able to influence the interaction between bFMO and indole substrate. Thus, site saturation mutagenesis was conducted to identify hotspot residues for improving the catalytic activity of bFMO. After screening the mutant libraries, positive mutants containing residues N291T, Q318L, S206N and D317S located in the substrate tunnel were found. The indirubin titers of recombinant *E. coli* strains containing mutants of N291T (60.1 ± 2.9 mg/L), Q318L (27.2 ± 4.3 mg/L), S206N (18.0 ± 2.0 mg/L) and D317S (22.6 ± 0.7 mg/L) were 5.0-, 2.3-, 1.6- and 1.9-fold higher than that of the control strain containing wild-type bFMO (11.9 ± 1.5 mg/L), respectively (Fig. 7a). The residual activity of mutants relative to wild-type bFMO is shown in Fig. 7b. The expression of N291T mutant was significantly lower than the wild-type bFMO and other mutants, indicating a higher specific activity of N291T mutant (Fig. S5). To further investigate the potential interactions of these positive residues, three combinatorial mutants of N291T/D317S, N291T/Q318L, and N291T/S206N were obtained by combined mutagenesis. The indirubin titers of recombinant *E. coli* strains containing the mutants N291T/D317S (24.1 ± 2.0 mg/L), N291T/Q318L (38.4 ± 3.2 mg/L), and N291T/S206N (13.8 ± 1.2 mg/L) were 2.0-, 3.2- and 1.2-fold higher than that of the control strain with wild-type bFMO (11.9 ± 1.5 mg/L), respectively, which was lower than that of the N291T mutant, indicating negative interactions among these residues (Additional file 1: Fig. S6).

Residue 291 is located on the flexible loop (residues 281–295) of bFMO, which is suggested to control the tunnel access that allows the substrate to enter the active pocket of bFMO (Fig. 2b). The N291T mutant exhibited a 1.9-fold (2.3 U/mg) increment in specific activity relative to that of native bFMO (1.2 U/mg). To gain deep insights into the mechanism underlying the improved catalytic activity of the N291T mutant, a comparative conformational analysis of native bFMO and the N291T mutant docked with indole was subsequently performed through molecular docking and molecular dynamic simulation. The comparison of the structure of the N291T mutant relative to the wild-type bFMO was performed, as shown in Fig. 7c, d. It was found that the carbonyl group of N291 generated hydrogen bonding with the hydroxy group of Y207 with a distance of 3.0 Å in wild-type bFMO, which constructed a tight substrate tunnel and could perform a pivotal role in determining the catalytic efficiency (Fig. 7c). The distance was increased from 3.0 Å for wild-type bFMO to 8.9 Å for the N291T mutant (Fig. 7d). Subsequently, the substrate tunnels of the wild-type bFMO and the N291T mutant were calculated with CAVER 3.0, and the detected diameter of the tunnel was widened from 2.4 Å in wild-type bFMO (Fig. 7e) to 4 Å in the N291T mutant (Fig. 7f).

The N291T mutant exhibited better catalytic performance with the increased *k*_cat_/*K*_m_ (0.45 s^−1^ mM^−1^) by 2.0-fold toward indole (Table 1). The *K*_m_ value of the N291T mutant toward indole was comparable with that of the wild-type bFMO. The *K*_m_ value of the N291T mutant toward NADPH decreased compared with that of wild-type bFMO, indicating more affinity toward NADPH (Additional file 1: Table S4). Structural analysis of the NADP(H)-bound form of native bFMO revealed that the substrate tunnel was occupied by NADP(H), which is located at a cleft interface between two domains (Alfieri et al. 2008). Thus, it was considered that NADP(H) was more easily entering with a looser substrate tunnel in the N291T mutant, which was beneficial for indole oxygenation reaction. Furthermore, N291S and N291A mutants with smaller residues were constructed (Additional file 1: Table S2). However, neither N291S nor N291A showed indole oxidation activity, and no product was detected, indicating that these mutations might destroy the structure of the original substrate tunnel and that proper expansion of the substrate channel was needed. Previous work has shown that the nicotinamide ring and the adjacent ribose of NADP^+^ are integrally engaged in the stabilization of the oxygenating intermediate (Alfieri et al. 2008). NADP(H) has a moonlighting role, which functions in both flavin reduction and oxygen reactivity modulation (Alfieri et al. 2008). Herein, we assumed that proper expansion of the substrate tunnel could be beneficial for indole and cofactor NADP(H) entering the active pocket and the enhanced oxygenation activity required for indirubin production.

**Table 1:** Steady-state kinetic parameters of bFMO and the mutants toward indole

Enzyme	Indole			
	*k*_cat_ (s^−1^)	*K*_m_ (mM)	*k*_cat_/*K*_m_ (s^−1^ mM^−1^)	Fold
WT	0.22 ± 0.01	0.98 ± 0.12	0.22 ± 0.02	1
N291T	0.45 ± 0.04	1.14 ± 0.30	0.45 ± 0.08	2.0
K223R	0.55 ± 0.04	0.75 ± 0.19	0.55 ± 0.08	2.5
K223R/D317S	0.79 ± 0.03	0.55 ± 0.14	1.45 ± 0.30	6.6
K223R/D317M	0.30 ± 0.03	1.61 ± 0.43	0.18 ± 0.03	0.8
K223R/D317A	0.16 ± 0.02	1.12 ± 0.31	0.14 ± 0.03	0.6

### Combined modification of the loop beside the *α*4 helix and the substrate tunnel

**Fig. 8:**
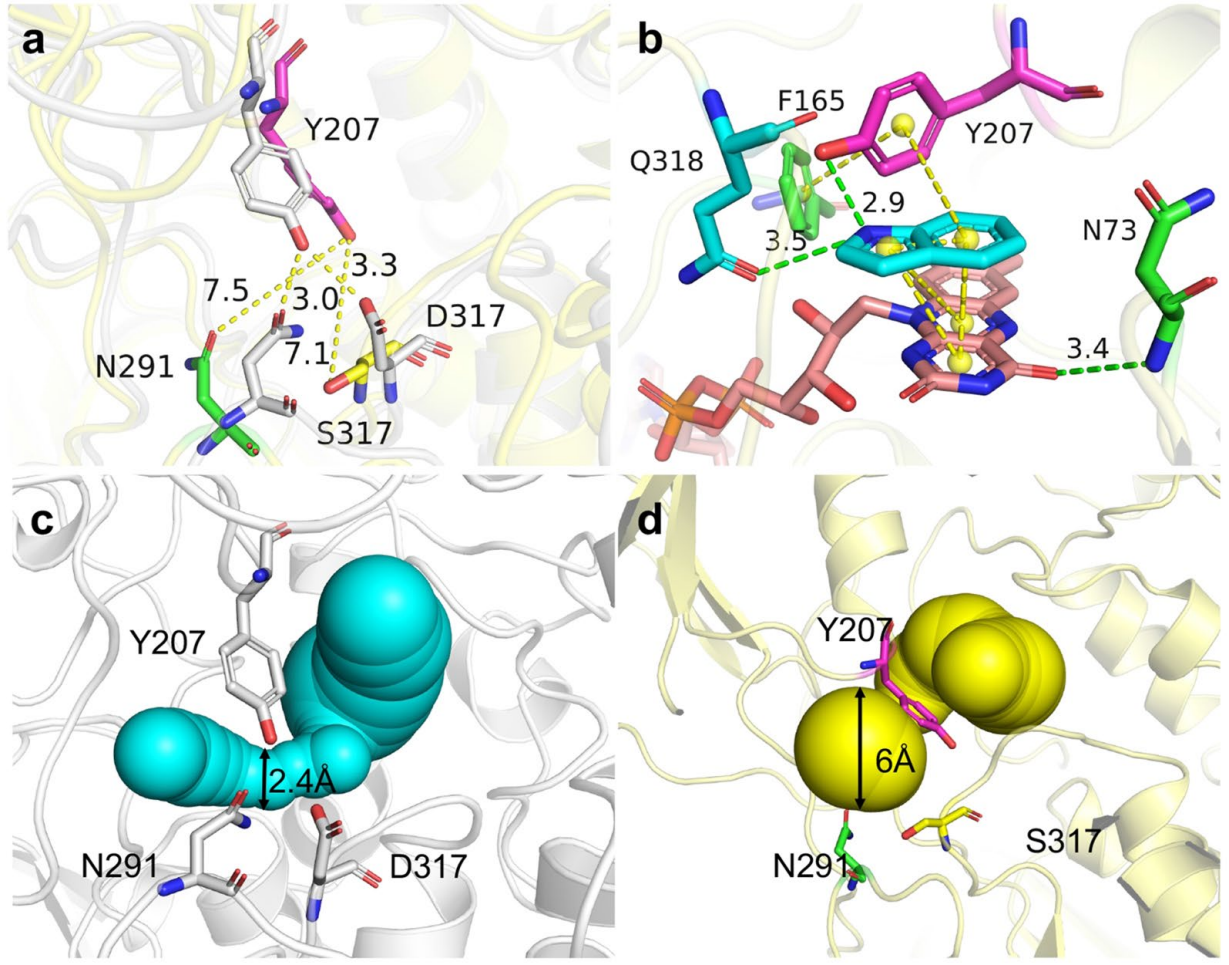
Structure of K223R/D317S mutant. **a** Comparison of the distance of residue 207–291 and residue 207–317 in wild-type bFMO (grey) and the K223R/D317S mutant (yellow), respectively. Residues are shown as gray sticks in wild-type bFMO. However, the residue 207, 291 and 317 are shown as purple, green and yellow sticks in the K223R/D317S mutant, respectively. **b** π–π interaction of Y207 (purple), indole (blue) and FAD (orange), and the π–π interaction between residues F165 and Y207 are shown in yellow dashed lines. The new hydrogen bond generated (green) between Y207 (purple) and Q318 (blue) to the indole (blue), respectively. The substrate tunnel shown in balls of wild-type bFMO (**c**) and the K223R/D317S mutant (**d**) was identified by CAVER 3.0

As shown in Fig. 8, it was assumed that the three residues Y207, N291 and D317 might play key roles in the substrate tunnel in bFMO. To further expand the substrate tunnel for potentially enhanced activity of bFMO, a double mutant, N291T/K223R, was constructed. The indirubin titer of the N291T/K223R mutant was 37.8 ± 2.9 mg/L, which was 2.9-fold higher than that of the control strain with wild-type bFMO, while it was lower than that of the N291T (60.1 ± 2.9 mg/L) and K223R mutants (76.8 ± 2.9 mg/L). We speculated that the combined mutation of N291T and K223R might result in conformational changes in bFMO, which might be disadvantageous for oxygenation activity, and the positive effects of N291T and K223R were nonadditive for oxygenation activity toward indole. A new hydrogen bond was generated between Y207 and D317 with a distance of 2.6 Å, which might lead to the loop (residues 204–208) connecting Y207 and the *α*4 helix being less flexible, thus stabilizing the *π*–*π* stacking interaction among the tyrosine ring of Y207, indole and the FAD cofactor (Fig. 6a). Molecular dynamics simulation indicated that the distance between Y207 and D317 was closer in the K223R mutant than in the wild-type bFMO. The relatively small distance between Y207 and D317 (2.6 Å) could constitute a tight substrate channel (Fig. 6c). Therefore, it was speculated that the entrance of substrate indole into the active pocket might still be the bottleneck in the K233R mutant.

Considering the newly generated hydrogen bonding and a relatively small distance between residues Y207 and D317, site saturation mutagenesis for the D317 residue based on the K223R mutant was further performed to improve the catalytic efficiency of bFMO. As a result, the indirubin titers of the K223R/D317S, K223R/D317M, K223R/D317A mutants in vivo were 113.4 mg/L, 44.9 mg/L and 34.1 mg/L, all of which were higher than that of the control strain containing wild-type bFMO. Only the K223R/D317S mutant showed higher indirubin production (1.5-fold) than the K223R mutant (76.8 mg/L). As shown in Table 1, kinetic analysis of the K223R/D317S mutant using indole substrate showed noticeably improved *k*_cat_/*K*_m_ of the K223R/D317S mutant (1.45 s^−1^ mM^−1^) and slightly decreased *k*_cat_/*K*_m_ of the K223R/D317M and K223R/D317A mutants (0.18 and 0.14 s^−1^ mM^−1^), compared with that of wild-type bFMO (0.22 s^−1^ mM^−1^), respectively. The K223R/D317S mutant significantly improved the affinity for the indole substrate, as indicated by the decreased *K*_m_ values (compared to the wild-type bFMO) in Table 1. The dynamic path of the substrate tunnel leading from the active site of a protein to its surface could greatly influence the behavior and properties of enzymes (Kreß et al. 2018). Nevertheless, there were no significant changes in the ratio of indigo and indirubin with the mutants compared to wild-type bFMO (Additional file 1: Table S3).

As shown in Fig. 8a, the diameter of the substrate channel ranges from approximately 3.0–4.0 Å in wild-type bFMO. In the K223R/D317S mutant, the distance between the tyrosine ring of Y207 and the amidogen groups of N291 and D317 was 7.5 Å and 7.1 Å, which was longer than that of the K223R mutant (4.7 Å and 2.6 Å), respectively. Y207, N291 and D317 are three key residues determining the diameter of the slot tunnel. The diameter of the substrate tunnel was enhanced from 2.4 Å for wild-type bFMO (Fig. 8c) to 6.0 Å for the K223R/D317S mutant (Fig. 8d), as determined by CAVER 3.0. Thus, the K223R/D317S mutation could enlarge the substrate tunnel and reduce steric hindrance, which accelerated the entrance of indole to the active pocket. A new hydrogen bond was generated between the amidogen group of Y207 and indole with a distance of 2.9 Å, while another hydrogen bond was generated between Q318 and indole, which could contribute to the stabilization of indole through polar interactions (Fig. 8b). Previous work has shown that subtle alterations in hydrogen bonding could drastically alter the efficiency and outcome of the reaction in FMOs. Meanwhile, the *π*–*π* stacking interaction between Y207 and indole also reinforced the stabilization of indole and the FAD cofactor. The dual roles of hydrogen bonding and *π*–*π* stacking interactions contributed to the stabilization of indole and the FAD cofactor, which was beneficial for improving the catalytic efficiency of bFMO for indirubin production.

Addressing highly flexible structural elements, such as loop regions and substrate tunnels, rather than focusing on the catalytic pocket of an enzyme, has attracted increasing attention in biocatalysts (Meng et al. 2021; Rapp et al. 2021). The potential of flexible loop and tunnel engineering to improve enzymatic functions is acknowledged (Kokkonen et al. 2019; Pavlova et al. 2009), but is experimentally still very underexploited in bFMO. Our results suggest that the double K223R/D317S mutant showed significant improvement in catalytic activity by combining the enlargement of the substrate tunnel and dual roles of hydrogen bonding and *π*–*π* stacking interactions induced by loop engineering for stabilization of the interaction of indole and the FAD cofactor, which provided an efficient strategy for improving the catalytic activity of FMOs.

### Efficient production of indirubin by an engineered *E. coli* strain

**Fig. 9:**
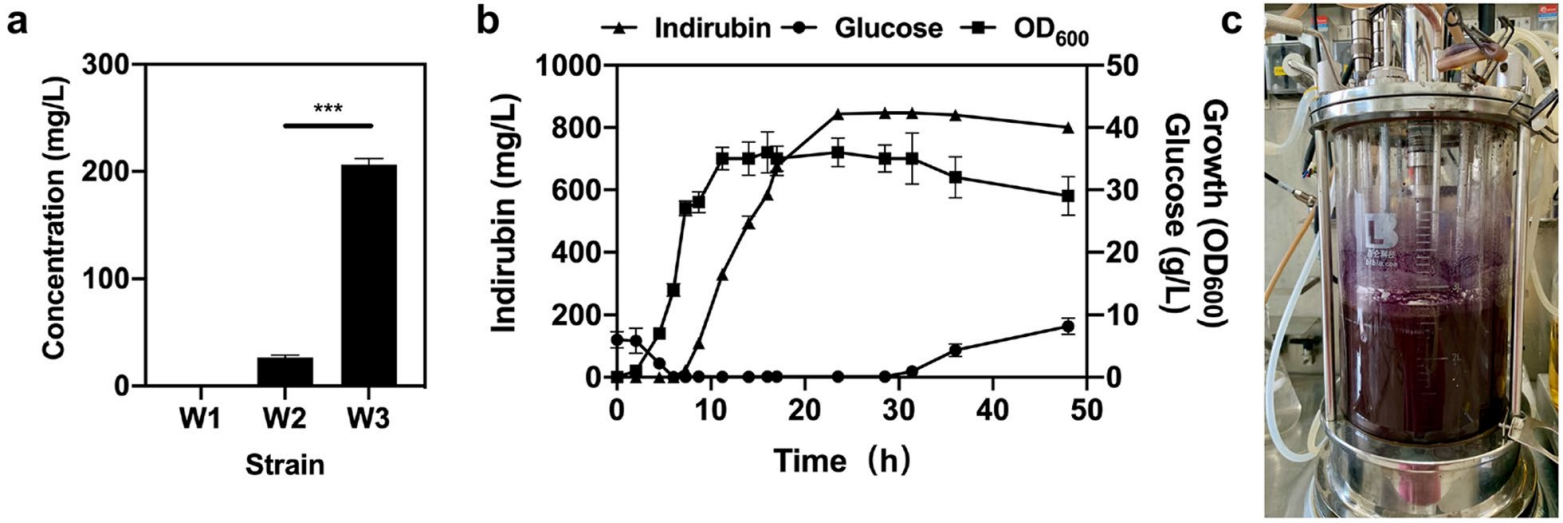
**a** Titer of indirubin in vivo of various mutants. **b** Fed-batch fermentation profiles of the engineered strain W3. Indirubin titer, cell growth and glucose concentration are shown. The data shown were chosen as a typical result from a series of experiments with consistent results. Error bar indicate standard error of the mean. **c** 5 L bioreactor used for fed-batch fermentation of the W3 strain

As a clear genetic background and well-established host, *Escherichia coli* is considered as one of the most promising chassis for biotechnology application. To construct an engineered strain with high yield of indirubin, we first attempted to develop a metabolically engineered *E. coli* based on the l-tryptophan (l-Trp) pathway. The pathway was divided into two modules: the inherent l-Trp pathway and heterologous pathway responsible for converting l-Trp to indirubin (Dickey et al. 2021; Du et al. 2018). To improve metabolic flux along the common aromatic amino acid pathway, the classic strain W1 for indirubin production was constructed by knocking out the genes *pykF* (encoding pyruvate kinase II), *pykA* (encoding pyruvate kinase I), *trpR* (encoding tryptophan repressor protein) and *ppc* (encoding phosphoenolpyruvate carboxykinase) of the competitive pathways using CRISPR–Cas9 system (Du et al. 2018), as shown in Additional file 1: Table S1. To investigate the effect of bFMO redesign on indirubin production in vivo, the gene *bFMO* was expressed under control of a constitutive promoter P_*tac*_ with plasmid pkk223-3, which was transformed to W1 to obtain strain W2 (Additional file 1: Table S1). With the similar strategy, successively, pkk223-3-FMO^K223R/D317S^ plasmid was transformed into W1 to obtain W3 strain (Additional file 1: Table S1). The titer of W3 was 206.4 mg/L, which was 7.8-fold higher than that of W2 (Fig. 9a). To comprehensively evaluate the overall indirubin production performance of W3, fed-batch fermentation was carried out in a 5 L bioreactor (Fig. 9c). As a result, the final indirubin titer was 860.7 mg/L, with a productivity of 18 mg/L/h in 48 h of fed-batch fermentation (Fig. 9b). To the best of our knowledge, this is the highest titer and productivity obtained for indirubin production in microbial organisms and was 3.43-fold higher than the maximum titer of indirubin reported thus far.

## Conclusions

In this work, a structure-guided semi-rational design was used to redesign bFMO for indirubin production, and molecular dynamics simulations were conducted to gain deeper insights into the dynamics of mutations with improved activities. A novel and efficient high-throughput FMO screening approach for the selection of positive mutants for indirubin production was constructed herein. Based on an in-depth understanding of the structure–function relationship of bFMO–substrate indole interactions, we have indicated that the flexibility of the key loop connected with conserved residues in the active pocket and the corresponding substrate tunnels are of high significance in bFMO, and several mutants were generated with improved catalytic performance to produce indirubin. Finally, we successfully developed an indirubin-producing strain with a high titer (860.7 mg/L) by improving the catalytic efficiency of bFMO in *E. coli*, which was 3.43-fold higher than the maximum production of indirubin ever reported thus far, and contributes to the progress for the efficient production of indirubin.

### Supplementary Information

**Additional file 1**: Experimental details Tables S1−2; The ratio of indirubin and indigo in the mutants (Table S3); Steady-state kinetic parameters of bFMO and the mutants toward NADPH (Table S4); Comparing the crystal structures of 2XVE and 2XVJ (Fig. S1); The 96-well-plants for screening the mutants (Fig. S2); The characterizations of the mutants (Figs. S3−S6).

## Data Availability

The data which this article is based upon are all included in this manuscript and the additional files associated with it.

## Abbreviations

bFMO: Flavin-containing monooxygenase
l-Trp: L-tryptophan
*pykF*: Pyruvate kinase II gene
*pykA*: Pyruvate kinase I gene
*trpR*: Tryptophan repressor protein gene
*ppc*: Phosphoenolpyruvate carboxykinase gene
