# Supplementary material for: Structure-guided engineering of a flavin-containing monooxygenase for the efficient production of indirubin
Source: Bioresour Bioprocess. 2022 Jun 27;9(1):70. doi: 10.1186/s40643-022-00559-7 (PMC10991670; doi:10.1186/s40643-022-00559-7)

Supporting Information

Structure-guided engineering of a flavin-containing monooxygenase for the efficient production of indirubin

Bing-Yao Sun, Hua-Lu Sui, Zi-Wei Liu, Xinyi Tao, Bei Gao, Ming Zhao, Yu-Shu Ma, Jian Zhao, Min Liu*, Feng-Qing Wang*, and Dong-Zhi Wei

1. State Key Lab of Bioreactor Engineering, Newworld Institute of Biotechnology, East China University of Science and Technology, Shanghai 200237, China.

^*^Address correspondence to

Min Liu: [lmin@ecust.edu.cn](mailto:lmin@ecust.edu.cn)

Feng-Qing Wang: [fqwang@ecust.edu.cn](mailto:fqwang@ecust.edu.cn)

E-mail addresses for other authors:

Bing-Yao Sun: sby18818278548@163.com

Hua-Lu Sui: shl18616321432@163.com

Zi-Wei Liu: Y30200483@mail.ecust.edu.cn

Xin-Yi Tao: xytao@ecust.edu.cn

Bei Gao: gaobei@ecust.edu.cn

Ming Zhao: zhaom@ecust.edu.cn

Yu-Shu Ma: myushu@ecust.edu.cn

Jian Zhao: [zhaojian@ecust.edu.cn](mailto:zhaojian@ecust.edu.cn)

Dong-Zhi Wei: dzhwei@ecust.edu.cn

Declarations of interest: none

Supplementary Information contents

**Table. S1** Strains and plasmids used in this study...............................................S-3

**Table. S2** Plasmids used in this study..................................................................S-4

**Table. S3** The radio of indigo and indirubin in the mutants................................S-5

**Table. S4** Steady-state kinetic parameters of bFMO and the mutants toward NADPH................................................................................................................S-6

**Fig. S1**............................................................................................................S-7

**Fig. S2**............................................................................................................S-8

**Fig. S3**............................................................................................................S-9

**Fig. S4**............................................................................................................S-10

**Fig. S5**............................................................................................................S-11

**Fig. S6**............................................................................................................S-12

**Additional Tables**

**Table S1. Strains and plasmids used in this study.**

| Names | Genotypes/Descriptions | Sources |
| --- | --- | --- |
| *Strains* |  |  |
| *E. coli* BL21(DE3) | host for protein expression | Stored in the lab |
| *E. coli* W3110 | wild type, the starting strain | Stored in the lab |
| W1 | *E. coli* W3110Δ*pykA*Δ*pykF*Δ*trpR*Δ*ppc* | This work |
| W2 | W1 containing pkk223-3-FMO | This work |
| W3 | W1 containing pkk223-3-FMO^K223R/D317S^ | This work |
| *Plasmids* |  |  |
| pET28a | Plasmid for overexpression, Kan^R^, T7 promoter | Stored in the lab |
| pET28a-FMO | Plasmid for FMO overexpression, Kan^R^, T7 promoter | This work |
| pET28a-FMO^K223R^ | Plasmid for FMO^K223R^ overexpression, Kan^R^, T7 promoter | This work |
| pET28a-FMO^N291T^ | Plasmid for FMO^N291T^ overexpression, Kan^R^, T7 promoter | This work |
| pET28a-FMO^K223R/D317S^ | Plasmid for FMO^K223R/D317S^ overexpression, Kan^R^, T7 promoter | This work |
| pET28a-FMO^K223R/D317M^ | Plasmid for FMO^K223R/D317M^ overexpression, Kan^R^, T7 promoter | This work |
| pET28a-FMO^K223R/D317A^ | Plasmid for FMO^K223R/D317A^ overexpression, Kan^R^, T7 promoter | This work |
| pkk223-3 | Plasmid for overexpression, Ap^R^, Ptac promoter | Stored in the lab |
| pkk223-3-FMO | Plasmid for FMO overexpression, ApR, Ptac promoter | This work |
| pkk223-3-FMO^K223R/D317S^ | Plasmid for FMO^K223R/D317S^ overexpression, ApR, Ptac promoter | This work |

**Tables S2. List of primers used for the construction of enzyme variants**

| **Mutation site** |  | **Sequence (5'to 3')** |
| --- | --- | --- |
| **47** | F | GATTGGGGCGGCCAGNNKAATTACACATGGCGC |
|  | R | GCCCCAATCAGCTTGTTTTTCAAAA |
| **66** | F | GGCGAACCTGTTCATAGCAGTNNKTATCGCTATCTGTGG |
|  | R | GCTATGAACAGGTTCGCCATT |
| **73** | F | CGCTATCTGTGGTCAAACNNKCCGAAAGAAT |
|  | R | CCACAGATAGCGATACATACTGCTA |
| **165** | F | TGTTGTACCGGTCACNNKTCAACACCTTACGTGCCT |
|  | R | ACCGGTACAACAGACAACATAGTCA |
| **205** | F | ACTGTATTACTGGTCGGCNNKAGTTACTCAGCTGAAGATATC |
|  | R | GCCGACCAGTAATACAGTTTTGTC |
| **206** | F | GTATTACTGGTCGGCAGCNNKTACTCAGCTGAAGAT |
|  | R | GCCGACCAGTAATACAGTTTTG |
| **207** | F | GTATTACTGGTCGGCAGCAGTNNKTCAGCTGAAGATATCGGCT |
|  | R | ACTGCTGCCGACCAGTAATACAGT |
| **208** | F | CTGGTCGGCAGCAGTTACNNKGCTGAAGATATCGGC |
|  | R | TAACTGCTGCCGACCAGTAATACA |
| **274** | F | ATTCTGTGTACCGGTNNKATCCATCACTTCCCC |
|  | R | ACCGGTACACAGAATAATCGCA |
| **291** | F | CTGCGTCTGGTCACCAATNNKCGTTTATGGCCGCTC |
|  | R | GGTGACCAGACGCAGATCGT |
| **291** | F | CTGCGTCTGGTCACCAATNNKCGTTTATGGCCGCTC |
|  | R | GGTGACCAGACGCAGATCGT |
| **291** | F | CTGCGTCTGGTCACCAATNNKCGTTTATGGCCGCTC |
|  | R | GGTGACCAGACGCAGATCGT |
| **317** | F | TACATTGGCATGCAGNNKCAATGGTACAGCTTC |
|  | R | CATGCCAATGTAGAAGAATTTTG |
| **318** | F | TACATTGGCATGCAGGATNNKTGGTACAGCTTCAAT |
|  | R | CTGCATGCCAATGTAGAAGAATTTT |
| **319** | F | ATTGGCATGCAGGATCAANKKTACAGCTTCAATATG |
|  | R | TTGATCCTGCATGCCAATGTAG |
| **220** | F | TCACAATGTTATAAATACNNKGCGAAAAAACTGATC |
|  | R | GTATTTATAACATTGTGAGCCG |
| **221** | F | CAATGTTATAAATACGGCNNKAAAAAACTGATCAGC |
|  | R | GCCGTATTTATAACATTGTGAGC |
| **222** | F | TGTTATAAATACGGCGCGNNKAAACTGATCAGCTGC |
|  | R | CGCGCCGTATTTATAACATTGT |
| **223** | F | TATAAATACGGCGCGAAANNKCTGATCAGCTGCTAC |
|  | R | TTTCGCGCCGTATTTATAACATT |

**S4**

**Table S3.** **The radio of indigo and indirubin in the mutants.**

|  | indigo (%) | indirubin (%) |
| --- | --- | --- |
| wt | 94.92 | 5.08 |
| N291T | 93.12 | 6.88 |
| K223R | 95.45 | 4.55 |
| K223R/D317S | 94.72 | 5.28 |
| K223R/D317M | 95.49 | 4.51 |
| K223R/D317A | 95.09 | 4.91 |

**Table S4. Steady-state kinetic parameters of bFMO and the mutants toward NADPH.**

|  | **NADPH** |  |  |
| --- | --- | --- | --- |
| **Enzyme** | ***k_cat_* (s^-1^)** | ***Km* (mM)** | ***k_cat_*/*Km*** |
|  |  |  | **(s^-1^mM^-1^)** |
| WT | 0.35 ± 0.02 | 0.10 ± 0.01 | 3.63 ± 0.34 |
| N291T | 0.55 ± 0.04 | 0.06 ± 0.01 | 8.96 ± 1.22 |
| K223R | 0.52 ± 0.05 | 0.07 ± 0.02 | 7.15 ± 1.41 |
| K223R/D317S | 0.47 ± 0.04 | 0.05 ± 0.01 | 9.22 ± 1.34 |
| K223R/D317M | 0.27 ± 0.03 | 0.08 ± 0.02 | 3.51 ± 0.45 |
| K223R/D317A | 0.13 ± 0.01 | 0.05 ± 0.01 | 2.43 ± 0.30 |

**Addition Figs**

**Fig. S1** By comparing the crystal structures of 2XVE and 2XVJ, the tyrosine ring of Y207 (red) in native bFMO occupies the space of substrate binding position (indole, gray) above the isoalloxazine ring of FAD (orange), thus preventing successful docking of indole into the active pocket of bFMO. Thus, N73 (blue), F165 (purple) and Y207 (red) were set as the flexible residues.


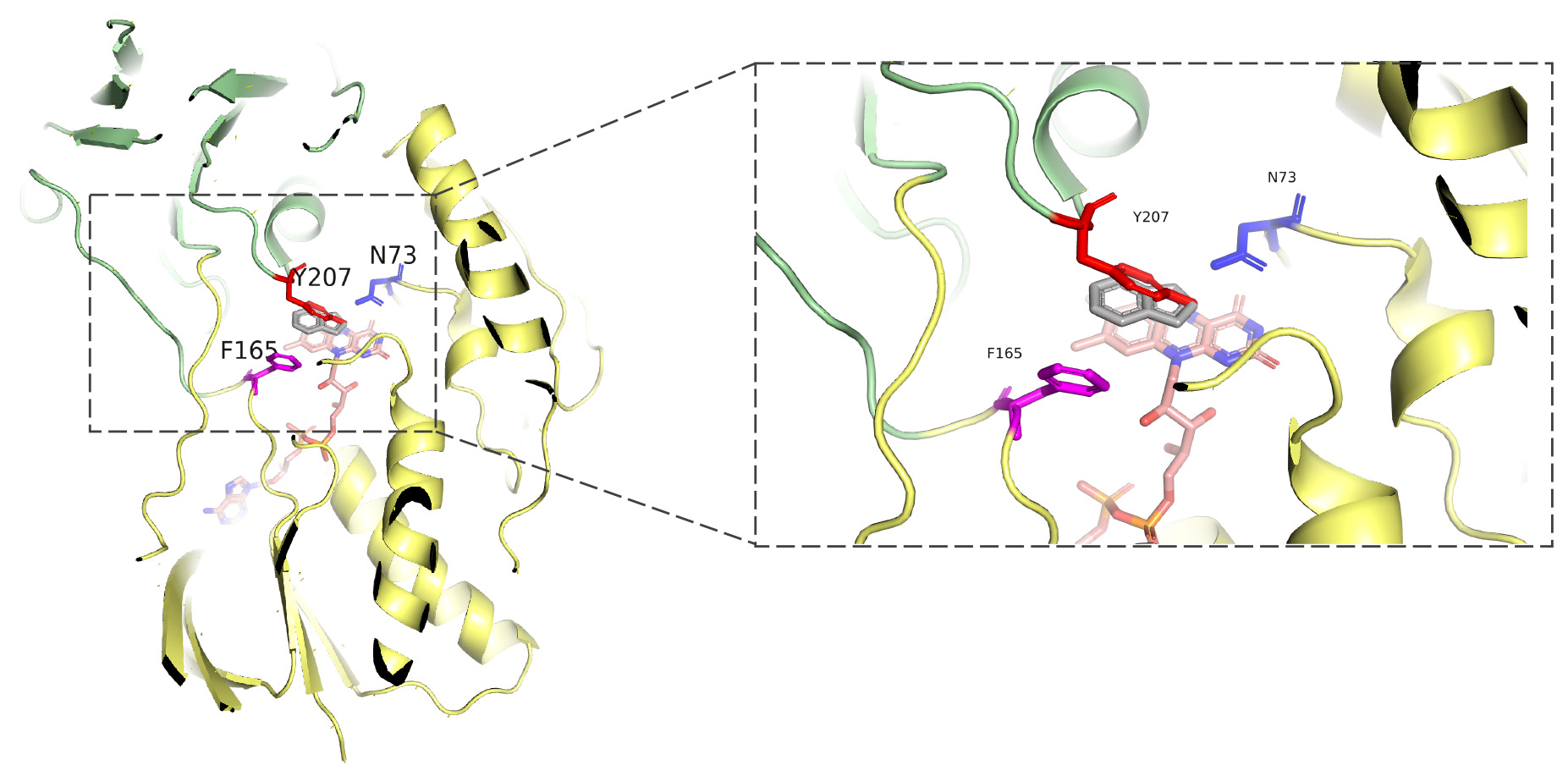


**Fig. S2** The 96-well-plants for screening the mutants from the saturated mutant library.

**
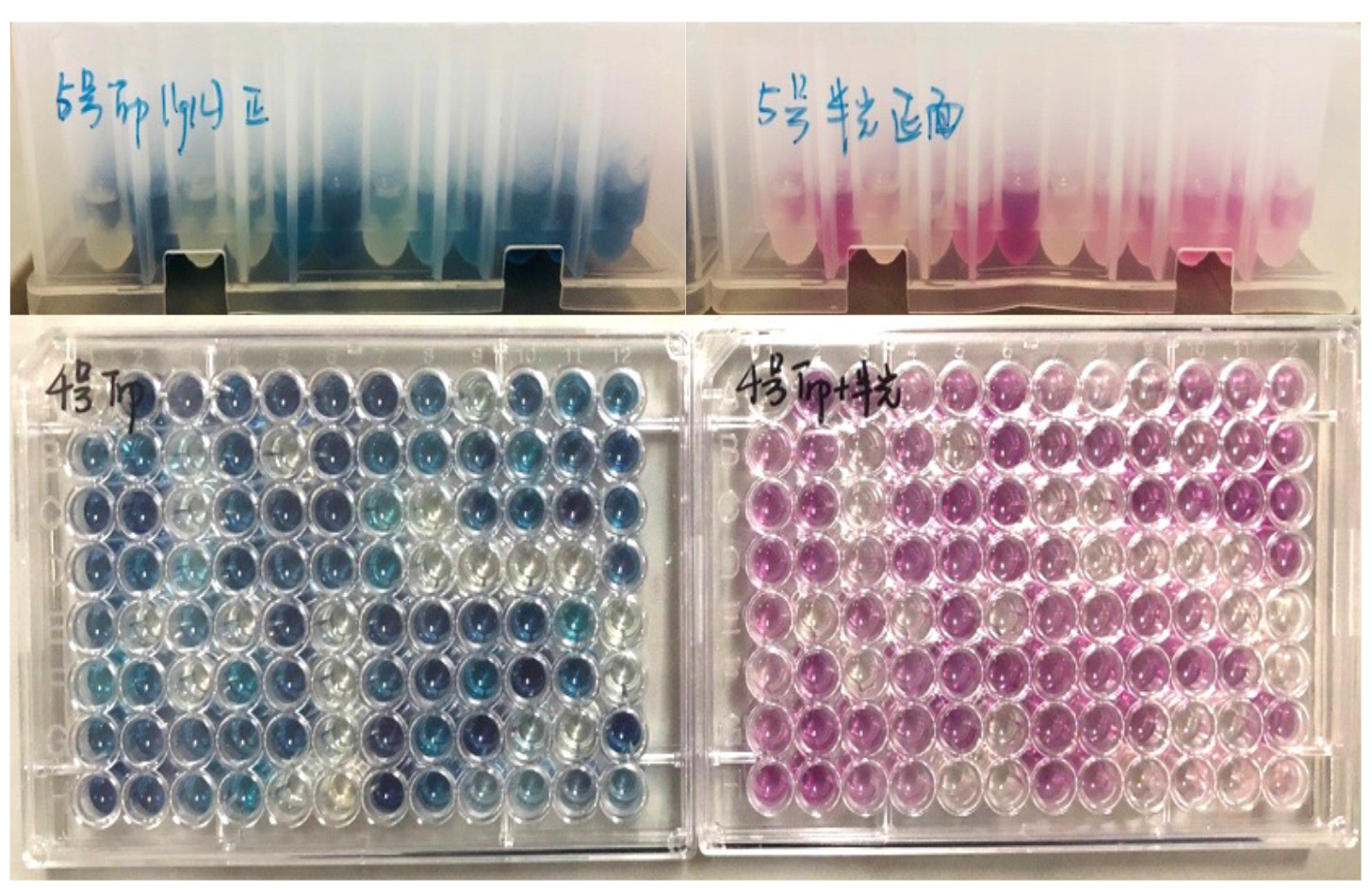
**

**Fig. S3** Kinetic study of wild-type bFMO and mutants N291T, K223R, K223R/D317S, K223R/D317M and K223R/D317A towards indole.

**
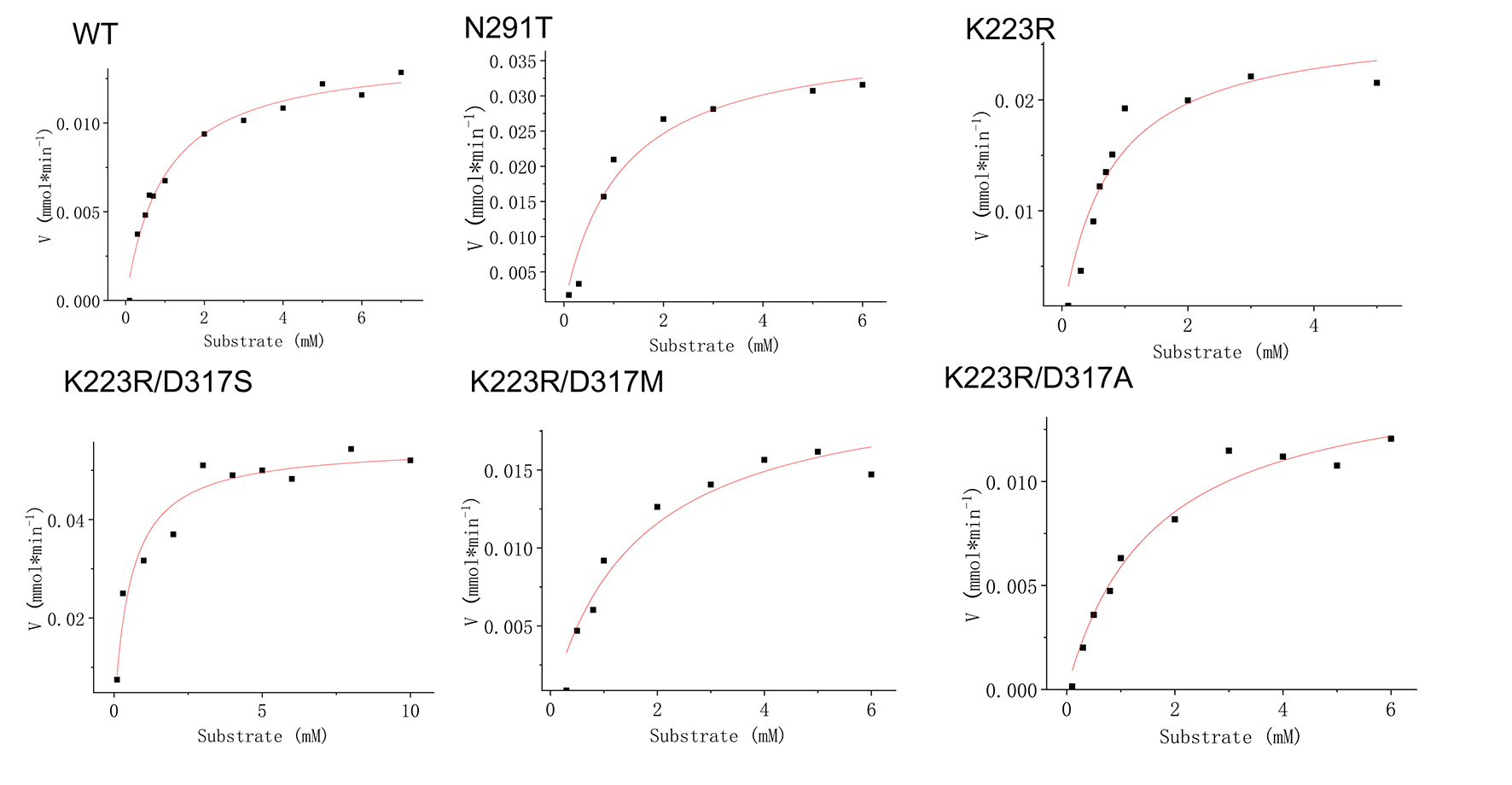
**

**Fig. S4** Residual activity of various mutants (residues 220-223) relative to the wide-type bFMO (1×).


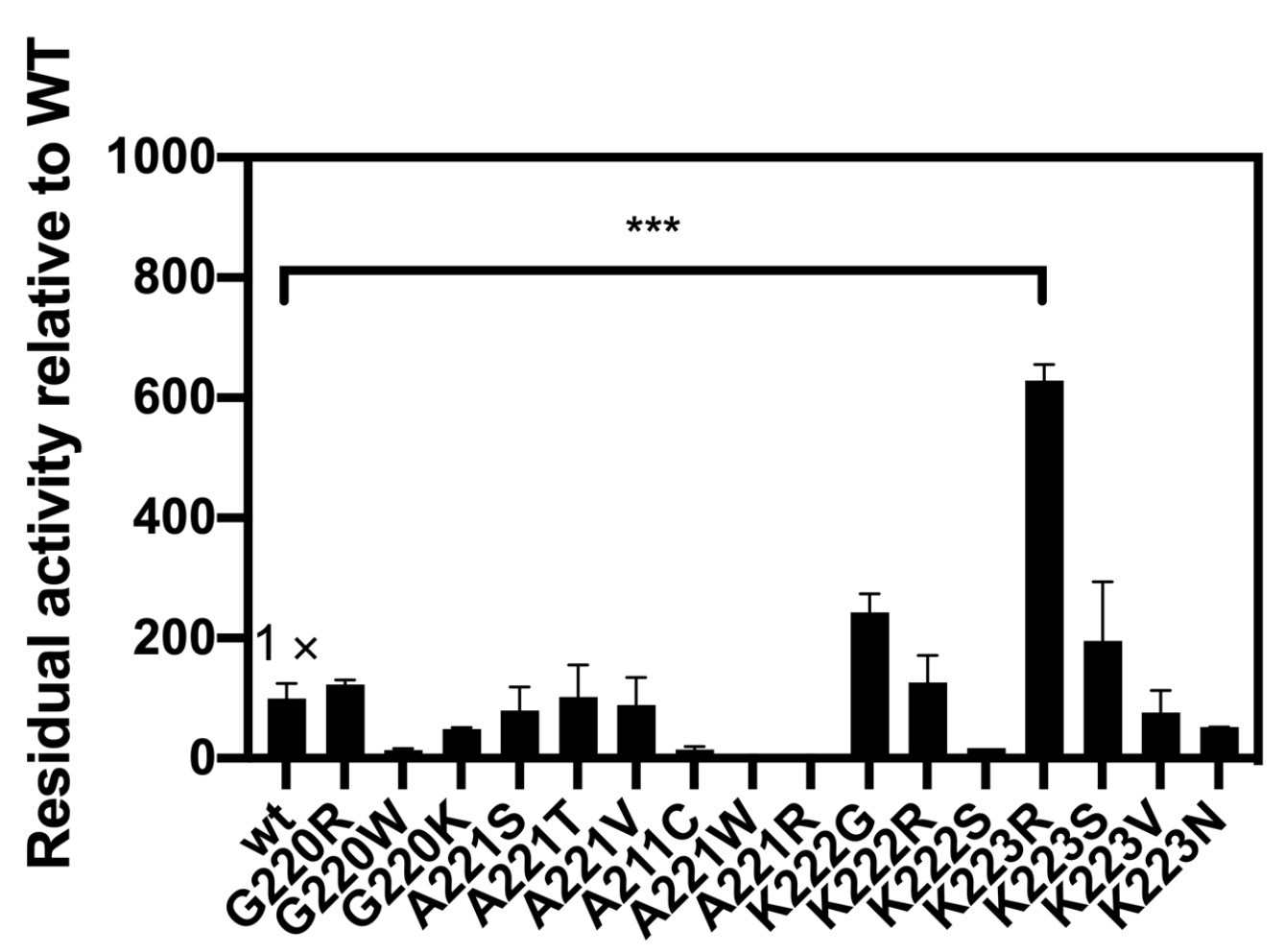


**Fig. S5** The SDS-PAGE analysis of wild-type bFMO and various mutants, respectively. The red box represented the bands of the bFMO protein.


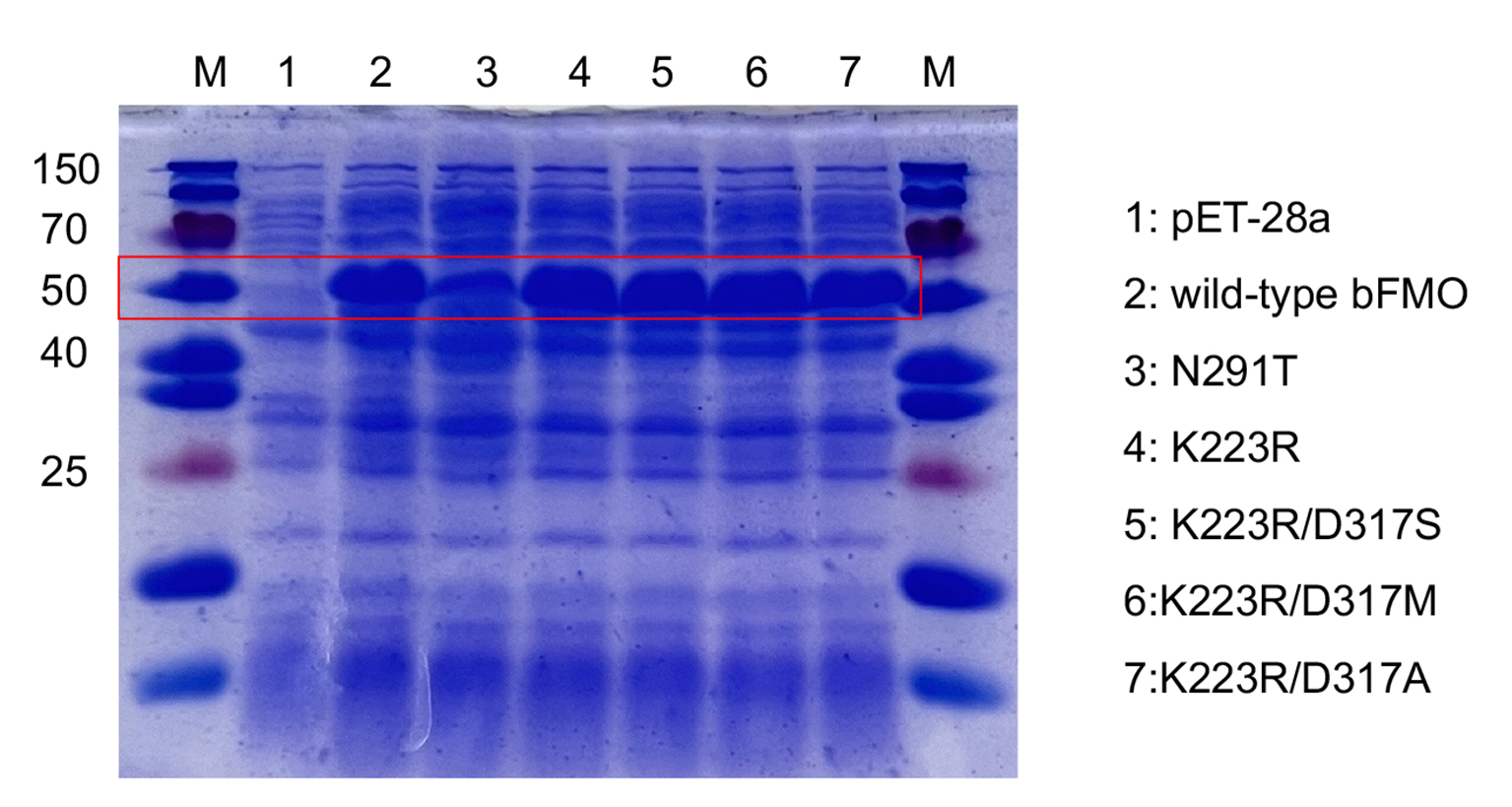


**Fig. S6** The indirubin titers of N291T/D317T, N291T/Q318L, and N291T/S206N mutants, respectively.


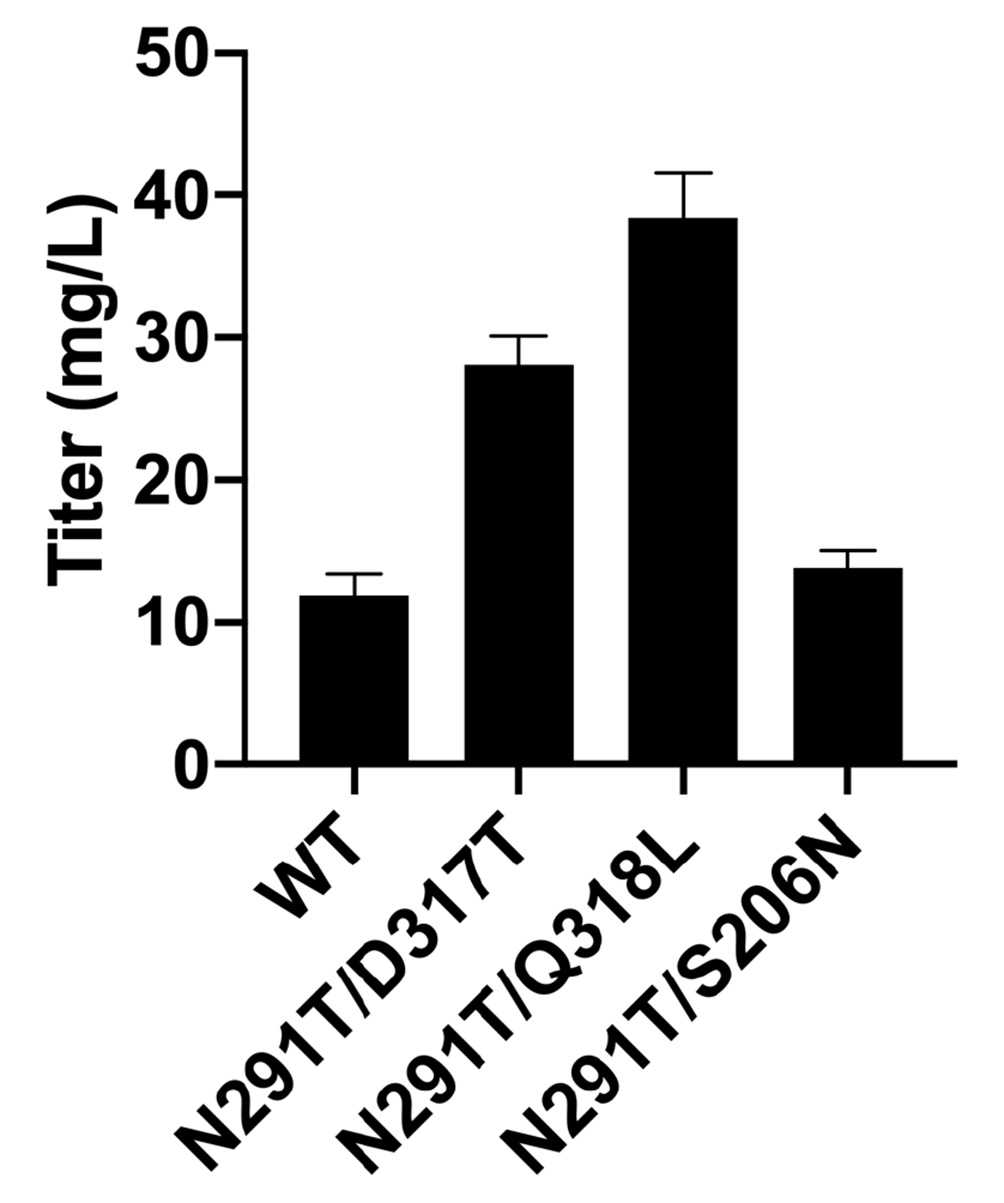

Supplement: Supplementary file 1 — Additional file 1: Experimental details Tables S1−2; The ratio of indirubin and indigo in the mutants (Table S3); Steady-state kinetic parameters of bFMO and the mutants toward NADPH (Table S4); Comparing the crystal structures of 2XVE and 2XVJ (Fig. S1); The 96-well-plants for screening the mutants (Fig. S2); The characterizations of the mutants (Figs. S3−S6). [file 40643_2022_559_MOESM1_ESM.docx]
